# Mapping the evidence on the impact of heat stress on exercise and work performance in females: a scoping review

**DOI:** 10.3389/fphys.2025.1507398

**Published:** 2025-06-03

**Authors:** Rachel E. Gilworth, Bethany D. Skinner, Daniel D. Hodgkiss, Samuel J. E. Lucas, Rebekah A. I. Lucas

**Affiliations:** School of Sport, Exercise and Rehabilitation Sciences, University of Birmingham, Birmingham, United Kingdom

**Keywords:** female, performance, productivity, heat, work, exercise

## Abstract

Increasing numbers of females are performing in increasingly hot environments. This scoping review aimed to 1) collate evidence on the effects of environmental heat stress on aerobic exercise performance and work productivity in females specifically, and 2) explore sex differences in the existing literature. A systematic search across four databases (PubMed, MEDLINE, Web of Science and SPORTDiscus) was developed based on MeSH terms and keywords, with all permutations relating to ‘FEMALE’, ‘WOMAN’, ‘HEAT’ AND ‘PERFORMANCE’. Identified articles were screened against pre-defined inclusion criteria related to age (16–60 years), environmental heat stress (≥23°C), and physical activity duration (≥5 min). We identified 35,696 articles, of which 41 met the inclusion criteria. Of the included studies, 19 reported female-specific comparisons, two of which also investigated sex differences. Four studies investigated sex differences alone, while 18 studies included females within the participant cohort. Thirty-eight of the included studies assessed athletic performance and three studies examined occupational performance (i.e., work output/productivity) in females. Existing data on the effect of heat stress on performance was predominantly from pre-menopausal cohorts (mean age 29 years, range 20–46 years), with no studies investigating peri- or post-menopausal cohorts. We uncovered limited research investigating the effect of menstrual cycle phase (six studies) or hormonal contraceptive use (two studies) on performance in the heat. Thirteen included studies examined interventions pre or during performance test(s), with four studies showing their interventions attenuated heat stress performance impairments in female cohorts. We highlight notable gaps in the literature regarding female performance in the heat; specifically, the influence of peri-post menopause, heat stress interventions for females, and impacts on females in the occupational sector. We recommend that researchers undertaking exercise and thermal physiological research aim for gender balance where possible and adhere to guidelines when designing and reporting research that encompasses females. Addressing these research gaps would provide workers, athletes, and practitioners with a better understanding of how to protect females and enhance their physical performance in the heat, across different stages of life, amidst a changing climate.

## 1 Introduction

Historically, it was perceived that strenuous physical activity compromised female health, in particular the female reproductive system (Gregg and Gregg, 2017). Women were also considered physically incapable of performing some military duties (Hemarajarajeswari and Gupta, 2021) and subsequently, were excluded from frontline combat roles. Such perceptions and barriers have delayed our understanding of the capability and capacity of females to perform strenuous activity. However, societal and governing authorities’ rules concerning female participation in endurance sport (e.g., long-distance running, marathons), the emergency services, and the military has evolved considerably over the past 30 years.

A major milestone for female inclusion in competitive sports occurred when females were first allowed to compete at the 1900 Paris Olympic Games, representing 2% of all competitors (Phulkar and Hardia, 20191). Another significant advancement came in 1972 when women were permitted to compete in the Boston Marathon. Since 2018, women have also been allowed to serve in all frontline military positions and now represent ∼12% of the UK and NATO armed forces (Kirk-Wade, 2024; NATO, 2020). These changes reflect broader societal shifts towards gender equality. Indeed, the 2024 Paris Olympics were the first Olympic Games to achieve equal representation of males and females across all sports. This was a historic achievement in athletic equality and showcases the exceptional performances of female athletes in recent years (IOC, 2024). Although the above milestones highlight advancements in female inclusion and participation in sports and the military, similar progress is needed in other sectors. For example, whilst women make up ∼43% of the global agricultural labour force (reaching as high as 80% in developing countries; (Raney et al., 2011; United Nations, 2015), significant gender inequalities persist in the access and control of resources (i.e., land, labour, information and technology; Sheahan and Barrett, 2014). This determines that women are disproportionally burdened by work-related heat stress in such settings, which contributes to productivity, income and health inequalities (Huyer, 2016; Sorensen et al., 2018). Thus, female participation in strenuous outdoor physical activity, which was traditionally male-dominated, has increased (Scheer, 2019). In other settings, females have long performed strenuous physical outdoor work/activity, with persistent gender inequalities increasing the risk/demand of such work (Patil and Babus, 2018).

Our climate is becoming increasingly challenging. Excessive heat stress exposure is an immediate impact of climate change, with days of extreme heat increasing in frequency and intensity alongside higher mean global temperatures (Romanello et al., 2022). For example, the 2020 Tokyo Olympic Games were the hottest recorded since 1952 (Yiou et al., 2023). Given the increasing acceptance of women’s proficiency in sports, military operations and other physically/thermally demanding occupations (Harvey, 2022), it is crucial to consider and address the effects of challenging environmental conditions on women’s performance capacity. Our current understanding of human thermoregulation and physical performance in the heat is largely based on data from predominately male cohorts (Hutchins et al., 2021). However, physiological, anatomical and endocrinal differences exist between males and females, meaning results may not be generalisable (Rich-Edwards et al., 2018). Females typically present with a smaller body size, higher surface area-to-mass ratio, higher relative fat content and lower aerobic fitness, all of which can affect heat storage (Havenith, 2001; Sharma and Kailashiya, 2016). Under high metabolic heat production rates, females also exhibit lower whole-body sudomotor activity compared to males (i.e., lower maximal sweat rate), even when heat production is matched (Gagnon and Kenny, 2011). Furthermore, the reproductive hormones oestrogen and progesterone vary across the menstrual cycle and from pre- to post-menopause, affecting thermoregulatory responses such as core body temperature (T_core_; Baker et al., 2020). Hence, in the context of human thermoregulation it is inappropriate to assume that females and males are analogues. Yet, how such biological sex differences affect performance under heat stress conditions remains poorly understood.

To advance research and practice for females in an increasingly warming world, a scoping review was conducted to map the available evidence regarding the effect of environmental heat stress on aerobic performance and work productivity in female-specific literature across the lifespan (i.e., females vs. females), with a secondary aim of examining sex differences in the literature (i.e., females vs. males).

## 2 Methods

### 2.1 Protocol and registration

This review was conducted using the Preferred Framework for Scoping Reviews (Arksey and O'Malley, 2005; Peters et al., 2015) and complies with the PRISMA-ScR checklist (Tricco et al., 2018). The final protocol was registered prospectively with the Open Science Framework on 19 February 2021 https://osf.io/r56sz/.

### 2.2 Information sources

The electronic databases PubMed, MEDLINE, Web of Science and SPORTDiscus were searched for all available publications from their respective inception to November 2023. Searches were limited to articles written in English language, without date restriction. The search strategy was developed in consensus with a librarian based on MeSH terms and specific terms using keywords, with all permutations relating to ‘FEMALE’, ‘WOMAN’, ‘HEAT’ and ‘PERFORMANCE’. The complete search strategy for each database is provided in the Supplementary Table S1. A manual search was performed of the reference lists of identified studies, and any retrieved systematic and narrative reviews to further identify relevant studies that were not captured within the database searches. Duplicates were screened out using the Covidence™ systematic review software (Veritas Health Innovation, Melbourne, Australia), before commencing the screening of abstracts and full texts.

### 2.3 Eligibility criteria

The inclusion and exclusion criteria were developed using the Population, Intervention, Comparison/Control Group, Outcome (PICO) framework (Higgins and Green, 2011). The criteria included population (e.g., healthy females, aged 16–60 years), intervention/domain studied (e.g., sustained physical activity/work/exercise lasting ≥5 min in duration or intermittent bouts lasting >30 s or 30 m in a heat stress environment until exhaustion or the maximal amount of work/exercise completed in a fixed amount of time) and outcome measure (e.g., the time to completion, work output, distance/duration). Studies included a control condition or control group comparison [e.g., temperate conditions, menstrual cycle phase, pre vs. post heat acclimation (HA), sex differences]. For the population criteria, a lower age limit of 16 years was selected as the legal age for work. The upper age limit of 60 years was selected as significant age-related declines in aerobic endurance and physical activity are observed in adults over 60 years (Hall et al., 2017). Wet Bulb Globe Temperatures (WBGT) of ≥ 23°C were considered a heat stress environment as this met the threshold for a high risk of heat-related illnesses according to the National Athletic Trainers’ Association (Binkley et al., 2002).

Study designs included randomised control trials (RCTs), pseudo RCTs, cross sectional studies, crossover studies, case control studies and observational studies. Individual case studies, conference abstracts, unpublished research and study protocols were excluded. Studies reporting: pregnant females; animals; water-based interventions or resistance-based exercise protocols measuring one repetition maximum; or exercise confined to the upper-body were excluded. Further detail of the PICO, selection and inclusion processes are outlined in the Supplementary Table S2.

### 2.4 Screening of abstracts and full texts

Seven researchers (RG, DH, BS, RL, EH, NB, NC) independently screened the literature, by first analysing titles and abstracts for relevance and eligibility criteria using Covidence™. Retrieved records were classified as included (yes), excluded (no), or uncertain (maybe). All evidence was screened by two researchers and any discrepancies were resolved by discussion and consensus between reviewers (RG, BS, RL).

**FIGURE 1:**
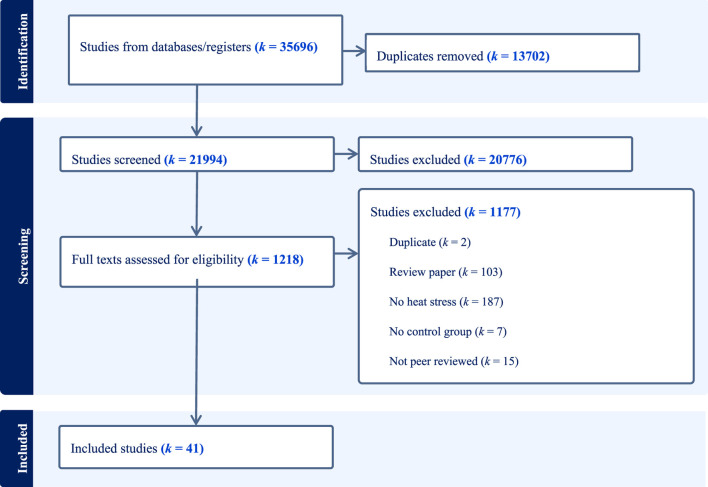
The PRISMA-P flow diagram for the scoping review detailing the database searches, the number of abstracts screened, and the full texts retrieved, where *k* denotes number of records.

Full-text articles were sourced and screened by five independent reviewers (RG, DH, BS, RL, MB), according to the eligibility criteria. Studies that met the inclusion criteria were included in the review and data was subsequently charted. Studies that did not meet the inclusion criteria at this stage, were excluded and the reason for exclusion was reported (Figure 1).

### 2.5 Data charting, level of evidence and quality assessment

Full texts that were deemed eligible for inclusion were charted independently by three reviewers (RG, BS and DH) using pre-tested data charting forms on Covidence™. All studies were charted in duplicate by two independent reviewers and were compared for consistency. If full texts could not be retrieved, or insufficient information was provided within a study, up to three attempts were made to contact the authors of articles which met the inclusion criteria.

### 2.6 Data items

Charted data included: study information, study characteristics, participant demographics, female characteristics, performance test, HA status, environmental conditions, details of interventions, performance outcomes, physiological outcomes and conclusions. The full data charting protocol can be found in the Supplementary File S1.

### 2.7 Critical appraisal of individual sources of evidence

The level and quality of evidence for each study was assessed in duplicate by independent reviewers (RG, BS, DH). Any disagreements were resolved through consensus with a fourth reviewer (RL). The level of evidence was deemed as High (level one; e.g., RCTs), Moderate (level two; e.g., cohort, case-control studies) or Low (level three; e.g., cross-sectional studies) using the criteria outlined in the Supplementary Table S3. To rate the quality of evidence for all studies we devised a modified 13 component tool based on the National Heart, Lung, and Blood Institute quality assessment tool for observational cohort and cross-sectional studies (Lung, 2014; Supplementary File S2). We independently rated each component as “Yes (2 points)”, “No (0 points)”, or “Partly (1 point)”. A study’s overall quality of evidence rating was deemed as High (26–39 points), Medium (12–25 points) or Low (0–13 points).

### 2.8 Synthesis of results

Where possible, results were charted as means of reported averages plus (range). Weighted means were calculated to account for differences in sample size between studies using the following formula: Σn*x̅/Σ*n*, where Σ = the sum of, *n* = number of participants in each study and, x̅ = mean outcome (e.g., age, mass, height, $\dot{V}$ O_2max_, ambient temperature, humidity and WBGT).

A narrative analysis was used to summarise the findings of the review. Studies reporting more than one valid comparison (e.g., females, hot condition vs. hot condition, and females, hot condition vs. temperate condition) have been reported more than once within the results or discussion section. Where papers included more than one valid environmental condition, the trial with the most extreme environmental condition was included. Throughout the review *k* denotes the number of studies, and *n* refers to number of participants. In the review, male and female terms refer to the biological sex attributes. The terms women and men are used more generally to refer to gender (Torgrimson and Minson, 2005). For studies that defined menstrual cycle phases as ‘early-’, ‘mid-’ and ‘late-’ luteal and follicular phase, these terms are used in the current review. Where studies have used the more general terms of ‘follicular’ and ‘luteal’ phase, these terms are used. Menstrual cycle phases are defined as quasi-follicular and quasi-luteal for OCP users. In the current review, naturally menstruating females have been defined as females who have a regular menstrual cycle lasting 21–35 days and have not used hormonal contraceptives for the past 3 months.

## 3 Results

### 3.1 Articles retrieved

**FIGURE 2:**
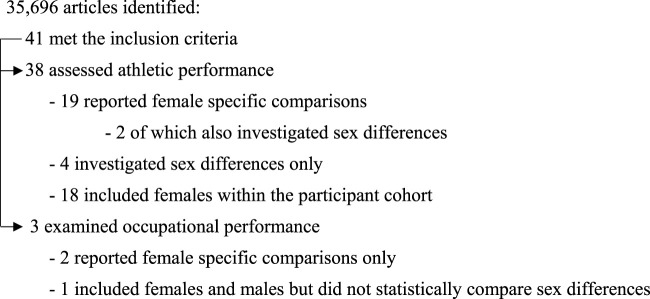
Schematic displaying an overview of all included studies, plus a breakdown of the athletic and occupational performance studies in the review.

A total of 35,696 citations were identified during the electronic database search (13,702 duplicates). A total of 21,994 articles were screened for title and abstract and 1,218 papers were eligible for full text screening (Figure 1). We contacted the authors of 71 papers for further data relating to our primary outcomes and seven authors responded with additional data. Overall, 41 studies were eligible for inclusion in this scoping review. A breakdown of the included aerobic performance-based studies and work performance studies are displayed in Figure 2. The main findings for all studies are summarised in Table 1 and a full overview of all performance data are reported in the Supplementary Table S4.

**TABLE 1:** Summary of performance outcomes and comparisons from all included studies (*k*=41).

Performance test	Total (*k*)	Participants (*n*, Females, Males)	Modality of performance test (*k*)	Athletic or work performance (*k*)	Types of comparisons
TT	29	822 (437, 385)	Running (12) Walking (3) Cycling (12) Rowing (2)	Athletic (28) Work (1)	Female vs. Female (13) Sex differences (4)** Female and male (13)
TTE	9*	147 (91, 56)*	Running (1) Walking (2) Cycling (6)	Athletic (9)	Female vs. Female (4) Sex differences (2)** Female and male (4)
$\dot{V}$ O_2max_ test	2*	29 (18, 11)*	Running (1) Cycling (1)	Athletic (2)	Female vs. Female (2) Sex differences (1)**
Productivity	2	30 (16,14)	Shoot harvesting (1) Work circuits (1)	Work (2)	Female vs. Female (1) Female and male (2)

*One study reported two performance tests ( $\dot{V}$ O_2max_ test and TTE) and subsequently has been reported twice. ** Three studies analysed female vs. female responses plus sex differences and subsequently have been reported twice in the table. Abbreviations: *k*, number of studies; *n*, number; TT, time trial; TTE, time to exhaustion; $\dot{V}$ O_2max_, maximal oxygen consumption. ‘Female and male’ refers to data that was not statistically compared.

### 3.2 Study characteristics

Studies were published from 1982 to 2023 with three published between 1982–1995, eight published between 1996–2010 and 30 published between 2011–2023. Nine were based in Asia, 11 in Oceania, 15 in North America, five in Europe and two in Africa. One of these compared race performance in Asia vs. Europe and hence was included twice. Of these studies, ten were RCTs, five were pseudo RCTs, one was a non-RCT, five were cohort studies, 14 were cross-sectional, one involved pre-post measures and five were case control studies.

### 3.3 Quality of the evidence

**FIGURE 3:**
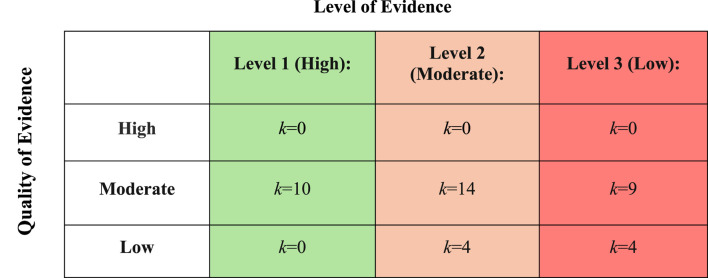
The level and quality of evidence for each included study (*k*=41).

10 studies were level one evidence, 18 studies were level two evidence, and 13 studies were classed as level three evidence. Using the modified 13-component tool based on the National Heart, Lung, and Blood Institute quality assessment tool (Lung, 2014), 66% of studies were rated as moderate quality and 33% were regarded as low quality evidence. None of the studies were rated as high quality (Figure 3).

### 3.4 Participant characteristics

**FIGURE 4:**
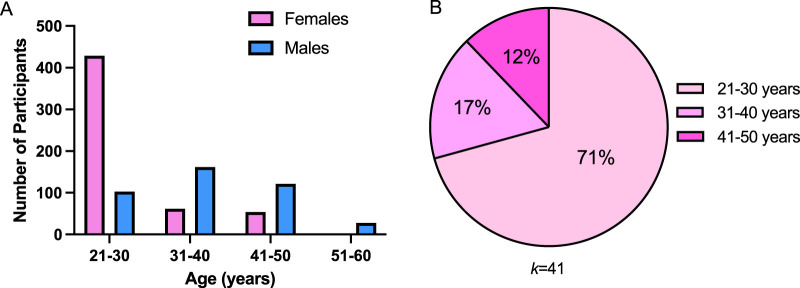
**(A)** represents mean distribution of the total number of female and male participants across all included studies. **(B)** represents age distribution of female participants across studies, *k* represents number of studies.

A total of 1,006 participants were reported across all studies. A summary of participant characteristics is provided in Table 2, while the distribution of ages and proportion of female participants’ age distribution are presented in Figures 4A,B, respectively. A detailed table of participant characteristics for all studies are provided in the Supplementary Table S4.

**TABLE 2:** Participant characteristics for all included studies, *n* represents number of participants. All other values are reported as weighted mean (range).

Characteristics	Females	Males
*n*	551	455
Age (years)	29 (20–46)	37 (22–54)
Mass (kg)	60.2 (40.1–73.0)	77.9 (57.4–92.2)
Height (cm)	165 (149–172)	179 (170–186)
V̇O_2max_ (mL/kg/min)	49 (35–60)	59 (44–69)

Of the included studies, eight investigated females who were naturally menstruating, eight investigated females using hormonal contraception, five included a mixed cohort of naturally menstruating and hormonal contraceptive users. Twenty-two studies did not report this information (see Supplementary Table S5 for further details; note that two studies are counted twice as they included separate cohorts of naturally menstruating females and hormonal contraceptive users). Of the included studies, 14 controlled for menstrual cycle, two reported the phase but did not control for menstrual cycle, and 25 did not report this information.

Overall, 21 studies controlled for the level of HA exposure prior to the performance test, by either employing a specific HA programme before the study took place or by ensuring participants were unacclimated at the start of the study. Two did not control for HA status and 18 studies did not report or were unable to control for HA status prior to testing.

### 3.5 Environmental conditions

Across all studies, mean ambient temperature for performance tests in hot conditions was 32.7°C (range 25.3°C–45.0°C) and mean RH was 57.8% (range 20.0%–83.0%), with this equating to a mean WBGT of 27.5°C (range 23.0–38.1). Eight studies had a temperate control that had a mean ambient temperature of 20.9°C (range 20.0°C–34.9°C) and mean RH of 51.6% (range 18.0%–25.0%), equating to a mean WBGT of 16.7°C (range 14.8°C–20.3°C). Further details on the environmental characteristics for all studies are provided in Supplementary Table S6. The environmental conditions of 24 studies were artificially manipulated (e.g., in an indoor environmental/climate chamber or with fans) and 17 studies were performed outdoors in natural conditions.

### 3.6 Performance measures

Across studies, various exercise modalities were utilised to assess performance. Five studies involved walking, 19 studies involved cycling, 13 studies involved running, two involved rowing, one involved work circuits and one involved plucking shoots. The type of performance assessment varied, with 29 using TT, nine using TTE, and two using $\dot{V}$ O_2max_ tests. Productivity was measured in two studies, which involved plucking shoots or completing work circuits.

### 3.7 Female vs. female comparisons

#### 3.7.1 Menstrual cycle phase

A higher basal T_core_ was reported during the luteal phase (*k* = 6). Four of the six identified studies reported no effect of menstrual cycle phase on prolonged exercise performance in hot conditions. Note, one of these studies included a mixed sample of naturally menstruating and hormonal contraceptive users. Two studies observed a performance decrement in the luteal phase as compared to the follicular phase despite no difference in final T_core_ between menstrual cycle phases (Table 3). Each study used calendar tracking to track menstrual cycle status, and five of six studies verified menstrual status with a resting blood sample to detect oestrogen and progesterone concentrations. Four of the six studies compared performance during the early-follicular phase (EF; day 3–6) to the mid-luteal phase (ML; day 19–25; Table 4).

**TABLE 3:** Thermoregulatory (basal, final and delta core body temperature) and performance outcomes for included studies investigating the effects of menstrual cycle phase on performance in hot conditions (*k*=6).

Study	Participant characteristics	Basal T_core_ (°C)	Basal T_sk_ (°C)	Final T_core_ (°C)	Delta T_core_ (°C)	Performance test	Performance outcome
Hashimoto et al. (2014)	$\dot{V}$ O_2max_ 39.5 ±5.3 mL/kg/min. Physically active (*n*=2); not physically active (*n*=4)	Elevated in L vs. F (P<0.05)	Not reported	Elevated in L vs. F (P<0.05)	Not reported	90-min cycling (50% V̇O_2peak_) followed by TT	NS
Lei 2017	$\dot{V}$ O_2max_ 55 ±9 mL/kg/min. Trained cyclists/triathletes (*n*=10)	0.21 ±0.14 **°**C higher in ML vs. EF (P<0.05)	NS	0.23 ±0.28 **°**C higher in ML vs. EF in hot humid (P<0.05) no difference in hot dry	Elevated in EF (1.40 ±0.18) vs. ML (1.23 ±0.27; P<0.05)	30-min cycling TT	NS
Wright 2002*	$\dot{V}$ O_2peak_ 51 ±1.9 mL/kg/min. Trained runners	Elevated in L (37.7) vs. F (37.42 ±0.28; P<0.05)	Not reported	NS	Elevated in F vs. L (P<0.05)	30-min running TT	NS
Zheng et al. (2021)	$\dot{V}$ O_2max_ 46.4 ±7.1 mL/kg/min	0.28 ±0.25 **°**C higher in ML vs. EF (P<0.05)	NS	NS	NS	20-min fixed-intensity cycling followed by 30-min TT	NS
Janse de Jonge 2012	$\dot{V}$ O_2max_ 40.0 ±6.9 mL/kg/min Recreationally active.	Elevated in ML (37.2 ±0.3) vs. EF (37.0 ±0.2; P<0.05)	NS	NS	Rest to 45-min was 19% higher in ML (P<0.05)	60-min fixed-intensity cycling (60% $\dot{V}$ O_2max_), followed TTE	TTE was 5.7% longer in EF than during ML (P<0.05)
Tenaglia 1999	$\dot{V}$ O_2max_ 44.8 ±2.6 mL/kg/min. Recreationally active.	Elevated in ML (37.31 ±0.08) vs. EF (37.09 ±0.08; P<0.05)	Elevated in ML (33.4 ±0.16) vs. EF (32.91 ±0.20; P<0.05)	NS	Elevated in EF vs. ML (P<0.05)	15-min walking, followed by 15-min seated rest until exhaustion	TTE was longer in EF (128.1 ±13.4 min) vs. ML (107.4 ±8.6 min; P<0.05)

Abbreviations: L, luteal; F, follicular; EF, early follicular; ML, mid luteal; T_core_, core body temperature; T_sk_, skin temperature; $\dot{V}$ O_2max_, maximal oxygen consumption; TTE, time to exhaustion; TT, time trial; NS, no significant difference between the menstrual cycle phases (P>0.05); mean ±SD. Delta T_core,_ difference between final and basal T_core_. * indicates the study used a mixed cohort of naturally menstruating and OCP users.

**TABLE 4:** Method and criteria for verifying menstrual cycle phase, and information on the menstrual cycle phase on the day of testing for all included studies investigating menstrual cycle effects on performance in hot conditions (*k*=6).

Study	Method of menstrual cycle phase verification		Criteria for menstrual cycle phase	Menstrual cycle phase during testing (day)	
	Calendar tracking	Oestrogen and progesterone concentration		Luteal (day)	Follicular (day)
Hashimoto 2014	Y	Y	Progesterone <5.1 ng/mL for luteal.	Exact day not specified. Concentrations of hormones are reported for each phase.	
Lei 2017	Y	Y	Progesterone > 9.5 nmol/L for ovulation.	ML (18 and 21)	EF (3 and 6)
Wright 2002	Y	N	None	Not reported	
Zheng et al. (2021)	Y	Y	Progesterone > 5 ng/mL for ovulation.	ML (20 ±2 and 22 ±3)	EF (3 ±1 and 7 ±2)
Janse de Jonge 2012	Y	Y	Progesterone > 16 nmol/L for luteal.	ML (19-25)	EF (3-6)
Tenaglia 1999	Y	Y	Progesterone level of > 9.5 nmol/L for ovulation.	ML (19-22)	EF (2-5)

Abbreviations: Y, yes; N, no; EF, early follicular; ML, mid luteal; mean ± SD.

#### 3.7.2 Hormonal contraceptives

Two studies investigated performance across the oral contraceptive pill (OCP) cycle in females using mono-phasic OCP. They found no difference in performance across the OCP cycle (Table 5).

**TABLE 5:** Reporting of oral contraceptive pill (OCP) characteristics, OCP taking phase, basal core body temperature, skin temperature and performance outcome for all included studies investigating OCP cycle effects on performance in hot conditions (*k*=2).

Study	Type of OCP	Brand, hormone concentrations in blood and synthetic hormone concentrations reported (Y/N)	OCP taking phase		Basal T_core_ (°C)	Basal T_sk_ (°C)	Final T_core_ (°C)	Performance test	Performance outcome
			qL (day)	qF (day)					
Lei 2019	Monophasic OCP (>1 year)	Y	qL (18–20 and 25–27)	qF (3–5 and 10–12)	Elevated in qL vs. qF (+0.15 ±0.21; P<0.05)	NS	NS	30-min cycling TT	NS
Tenaglia 1999	Monophasic OCP (*n* = 7); triphasic (*n* = 2).	Y	qML (19±22)	qEF (2±5)	Elevated in qL (37.14) vs. qF (37.36; P<0.05)	NS	NS	15-min walking, followed by 15-min seated rest until exhaustion	NS

Abbreviations: Y, yes; N, No; qL, quasi-luteal; qF, quasi-follicular; qEF, quasi-early follicular; qML, quasi-mid luteal; OCP, oral contraceptive pill; T_core_, core body temperature; T_sk_, skin temperature; TT, time trial; NS, no significant difference between the OCP taking phases (P > 0.05).

#### 3.7.3 Naturally menstruating females vs. hormonal contraceptive users

Three studies assessed the effects of heat stress on performance in naturally menstruating females as compared to hormonal contraceptive users taking monophasic or triphasic (Tenaglia et al., 1999), monophasic only (Lei et al., 2019) or triphasic only (Sims et al., 2007) OCPs. Two studies found no discernible differences in performance between naturally menstruating females (matched for height, mass and fitness characteristics) and OCP users (Sims et al., 2007; Lei et al., 2019). One study found performance was impaired during the ML phase compared to the EF phase for naturally menstruating females, but was not different between the quasi-ML and quasi-EF in OCP users (Tenaglia et al., 1999).

#### 3.7.4 Work productivity

One study investigated work-rest ratios during moderate intensity exercise in uncompensable heat stress with a mixed sample of OCP users and naturally menstruating females and found no difference across the menstrual/OCP cycle (Tenaglia et al., 1999). Another study investigated repeated work circuits and included females in their participant cohort (Vincent et al., 2018). The third occupation-focused study observed work productivity in ‘slow’ (n = 3) vs. ‘fast’ (n = 3) female workers (Sen et al., 1983).

#### 3.7.5 Environmental conditions

Four studies found that prolonged exercise performance was impaired in hot as compared to temperate conditions. One study found no effect of ambient temperature on performance and one study did not statistically analyse the data. Two studies reported that prolonged exercise performance was impaired in hot, wet conditions compared to hot, dry conditions (Table 6).

**TABLE 6:** Environmental conditions, performance protocol, performance outcome and percentage change between environmental conditions for all studies investigating female performance in hot compared to temperate conditions (*k*= 6) or hot wet vs. hot dry conditions (*k*=2).

Study	Environmental conditions			Performance test	Performance		Percentage change between conditions
	Temperature (°C)	Humidity (%)	WBGT (°C)				
Taylor 2014	Hot: 35	60	30	2000 m TT	Time, Hot: 9:0 ±0:3 min	Mean PO, Hot: 133 ±6 W	PO and completion time were not different between Hot and Temp (P>0.05)
	Temp: 22	39	16		Time, Temp: 8.5 ±0.3 min	Mean PO, Temp: 141 ±5 W	
Slater 2005	Hot: 32	60	28	1200 m TT	Time, females Hot_Fluid restriction_: 461.4 ±11.2 s Hot_No-fluid restriction_: 457.9 ±10.1 s	Time, males Hot_Fluid restriction_:403.3 ±7.8 s Hot_No-fluid restriction_: 403.0 ±6.0 s	Performance was impaired by 1% in Hot vs. Temp (P<0.05)
	Temp: 21	29	15		Time, females Temp_Fluid restriction_: 457.2 ±9.3 s Temp_No-fluid restriction_: 453.7 ±10.0 s	Time, males Temp_Fluid restriction_: 400.3 ±7.4 s Temp_No-fluid restriction_: 398.2 ±7.4 s	
Zheng et al. (2021)	Hot: 32	53	29	20-min fixed intensity followed by 30-min TT	Work, Hot: 272 ± 9 kJ	Mean PO, Hot: 151 ±33 W	PO and total work impaired by 3% in Hot vs. Temp (P<0.05)
	Temp: 20	75	16		Work, Temp: 280 ±57 kJ	Mean PO, Temp: 156 ±32 W	
Janse de Jonge 2012	Hot: 32	60	28	60-min fixed intensity followed by TTE	Time, Hot: Luteal 63.6 ±10.4 min vs. Follicular 66.7 ±8.1 min		TTE impaired by 10% in Hot vs. Temp (not statistically compared)
	Temp: 20	45	15		Time, Temp: Luteal 71.9 ±4.2 min vs. Follicular 72.8 ±3.0 min		
Ftaiti 2010	Hot: 35	59	30	60% maximal aerobic power TTE	Time, Hot: 46.4 ±10 min		TTE impaired by 34% in Hot vs. Temp (P<0.05)
	Temp: 22	53	18		Time, Temp: 70.4 ±14 min		
Arngrimsson 2014b	Hot: 45	50	38	TTE and $\dot{V}$ O_2max_	Time, females Hot: 9.1 ±1.8 min	Time, males Hot: 8.77 ±1.31 min	TTE impaired by 28% in Hot vs. Temp (P<0.05). Hot impairment similar for males and females in $\dot{V}$ O_2max_ and TTE
	Temp: 24	50	20		Time, females Temp: 12.6 ±1.5 min	Time, males Temp: 13.15 ±1.41 min	
Beal et al. (2022)	Hot: 32	78	30	Marathon running race	Pace, Hot: 14.8 ± 1.0 km/h		Pacing 5% slower in Hot vs. Temp (P<0.05)
	Temp: 19	56	16		Pace, Temp: 15.5 ± 1.0 km/h		
Lei 2017	Hot: 34	42	27	30-min cycling TT	Work output, Hot_Dry_: 263 ± 39 kJ		Performance impaired by 7% in Hot_Humid_ vs. Hot_Dry_ (naturally menstruating females; P<0.05)
	Hot: 29	83	27		Work output, Hot_Humid_: 248 ± 40 kJ		
Lei 2019	Hot: 34	41	27	30-min cycling TT	Work output, Hot_Dry_: 273 ±29 kJ		Performance impaired by 7% in Hot_Humid_ vs. Hot_Dry_ (naturally menstruating females; P<0.05)
	Hot: 29	81	27		Work output, Hot_Humid_: 258 ±28 kJ		Performance impaired by 7% in Hot_Humid_ vs. Hot_Dry_ (OCP users; P<0.05)

Abbreviations: Temp, temperate; WBGT, Wet Bulb Globe Temperature; PO, power output, W, Watts; $\dot{V}$ O_2max_, maximal oxygen consumption; TT, time trial; TTE, time to exhaustion; min, minutes; s, seconds; OCP, oral contraceptive pill. Data is for females unless stated otherwise.

#### 3.7.6 Interventions

Of the 41 studies, 13 included an intervention pre or during performance test(s). Nine studies specifically investigated the effect of an intervention on female performance in hot conditions (Table 7) and four studies included females as part of the participant cohort, but did not specifically compare female-specific data or sex differences (Hunter et al., 2006; Arngrïmsson et al., 2004a; Casa et al., 2010; Slater et al., 2005). Of the studies specifically investigating females, one study reported that pre-cooling had no effect on final T_core_ or performance compared to control conditions (*k* = 2), despite a lower basal T_core_ reported in one study. Consuming a carbohydrate beverage (*k* = 1) or caffeine (*k* = 1) had no effect on prolonged exercise performance in the heat compared to placebo. Long-term HA (9-days; *k* = 1), a pharmaceutical (Bupropion, *k* = 1), a high sodium beverage (*k* = 1) and menthol mouth rinse (*k* = 1) improved female performance in hot conditions compared to the control trial (P < 0.05). Pre-heating (*k* = 1) impaired female performance in hot conditions, relative to no pre-heating.

**TABLE 7:** Type of intervention acute/chronic, performance test and performance outcome of all included studies investigating the effect of an intervention on female vs. female comparisons using hot vs. hot conditions (*k*=9).

Study	Type of intervention	Intervention	Acute/chronic	Performance test	Basal T_core_ (°C)	Final T_core_ (°C)	Performance change
Zimmermann 2017	Cooling	30-min pre-cooling with ice vs. water	Acute	800 kJ cycling TT	Lower after pre-cooling vs. water (P<0.05)	NS	NS
Taylor 2014		20-min pre-cooling (cold shower or rest)	Acute	2,000-m rowing TT	NS		NS
Arngrimsson 2014b	Pre-heating	20-min exercise induced pre-heating vs. no pre-heating	Acute	$\dot{V}$ O_2max_ test	Basal T_core_ and final T_core_ elevated after pre-heating vs. no pre-heating (P<0.05)		Pre-heating impaired performance (11.4 ±1.4 vs. 9.0 ±1.8 min; P<0.05)
Gavel et al. (2021)	Nutrition	Menthol mouth rinse vs. placebo	Acute	30 km cycling TT	NS		Menthol mouth rinse improved TT performance (2.3%) and PO (P<0.05)
Hashimoto 2014		Carbohydrate vs. placebo beverage	Acute	90-min cycling (50% V̇O_2peak_) followed by TT	NS		NS
Suvi 2017		Caffeine vs. placebo	Acute	Walk to exhaustion	NS		NS
Sims 2007		High sodium vs. low sodium beverage	Acute	Cycling 70% V̇O_2peak_ until exhaustion	Delta T_core_ greater in the low sodium group vs. high sodium group		TTE 21% improvement in high sodium (98.8 ±25.6 min) vs. low sodium (78.7 ±24.6 min, P<0.05)
Cordery et al. (2017)	Pharmaceutical	Bupropion vs. placebo	Acute	60-min cycling at 60% V̇O_2peak_ followed by a 30-min TT	NS	Elevated in bupropium vs. placebo (P<0.05)	Bupropion increased work (291 ±48 kJ) vs. placebo (269 ±46 kJ, P<0.05).
Kirby 2019	HA	9- days HA	Chronic	15-min cycling TT	NS		9-days HA improved distance (3%), PO (8%) and speed (3%; P<0.05)

Abbreviations: T_core_; Core body temperature; TT, time trial; HA, Heat acclimation; WBGT, Wet Bulb Globe Temperature; PO, power output; kJ, kilojoule; V̇O_2peak_ maximal oxygen consumption at peak; min, minutes; s, seconds. NS, no significant difference between intervention and control (P > 0.05).

### 3.8 Female vs. male

Six studies formally investigated the effect of biological sex on performance in hot conditions. Twelve studies reported male and female performance outcome data separately but did not statistically compare male vs. female performance in hot conditions. Only one study investigated sex differences in response to an intervention, specifically caffeine ingestion (Suvi et al., 2017), which had no effect on performance in either sex. Three studies compared performance under hot conditions between females and males (Armstrong et al., 2012; Hosokawa et al., 2016; Wright et al., 2002), two studies found no significant difference between sexes (Armstrong et al., 2012; Hosokawa et al., 2016) and one study found males were significantly faster than females (O’Neal et al., 2012). One study investigated the effect of hyperthermia on $\dot{V}$ O_2max_ and TTE in males and females and found the reduction in $\dot{V}$ O_2max_ and performance to be similar between the sexes (Arngrímsson et al., 2004b).

## 4 Discussion

### 4.1 Main findings

To our knowledge, this is the first scoping review to map the available literature investigating the effects of environmental heat stress on aerobic performance and work productivity in females. Despite an extensive systematic review of the literature, only 41 studies met our inclusion criteria (i.e., included healthy adult females and examined performance during sustained physical activity/work/exercise in a hot (WBGT ≥ 23°C) environment). Thus, there remains an alarmingly limited number of heat stress and performance-based studies with females included in their study cohort(s). Of these 41 studies, only 19 examined biological sex differences or female-specific performance in the heat. Most notably, there were very few (i.e., *k* = 3) occupational performance studies and no studies examining performance in peri- and post-menopausal females (i.e., aged > 40 years). Furthermore, few studies have investigated the effect of interventions on female performance in the heat. Consequently, this scoping review has identified some key areas for future research.

### 4.2 Study characteristics

A recent comprehensive systematic review by Hutchins et al. (2021) has highlighted the significant underrepresentation of females in exercise thermoregulation literature over the past decade, with females accounting for only 30% of total participants in 2019. The current systematic scoping review echoes this finding, as only 41 studies met the inclusion criteria. This highlights a serious dearth of literature including females, either as part of or forming an independent cohort, when examining physical performance in hot environments. It is noteworthy that 90% of the included studies for the current review were conducted after the year 2000, indicating a promising increase in the inclusion of female cohorts over the last ∼25 years. We recognise this is a step forward and an important move towards addressing gender inequity in the scientific evidence and guidance available to females performing in such contexts.

### 4.3 Performance: female vs. female

#### 4.3.1 Environmental conditions

In hot conditions, marked performance decrements differences were reported across studies (ranging from no observed differences to 34%) when compared to temperate conditions. These discrepancies likely result from differences in the duration and intensity of performance protocols as well as the environmental conditions in which the experimental trials were conducted. Studies that reported the greatest reductions in performance (20%–34%) involved either an incremental $\dot{V}$ O_2max_ test (Arngrímsson et al., 2004b) or a moderate exercise intensity TTE test (Ftaiti et al., 2010). Additionally, dry heat as compared to humid heat, was associated with significant performance impairments in females when WBGT was matched (Lei et al., 2019; Lei et al., 2017).

#### 4.3.2 Menstrual cycle

Only two of the six included studies examining the effect of menstrual cycle phase reported that luteal-phase-related elevations in T_core_ impaired aerobic performance (Tenaglia et al., 1999; Janse de Jonge et al., 2012). These studies were conducted under conditions of high humidity or uncompensable heat stress, where the evaporative capacity for heat loss was reduced. At present there is limited evidence to suggest naturally menstruating females should be advised to adjust their competition schedule based on their menstrual cycle.

In total, 5/6 studies investigating the effect of the menstrual cycle on performance were conducted in accordance with current guidelines to determine menstrual cycle phase (i.e., reporting exact day of testing within phase, verified by resting blood samples to quantify oestrogen and progesterone levels; Janse de Jonge et al., 2019). Notably, 4/6 reported the concentration of oestrogen and progesterone at the time of testing. However, there were discrepancies in how these studies applied post-ovulatory progesterone criteria. This calls to attention the broader debate on the most appropriate progesterone threshold value for determining ovulation in pre-menopausal female participants (Landgren et al., 1980; Israel et al., 1972).

Given the potential physiological impacts of menstrual cycle phase on thermoregulation (e.g., on basal T_core_ and thermo-sensitivity; Janse de Jonge et al., 2012; Kuwahara et al., 2005), it is concerning that almost half of the performance-focused studies in this review did not report participants’ menstrual cycle phase during testing. Experimental design may prevent or mitigate the need to control for menstrual cycle phase (i.e., observational field studies within occupational or athletic settings or repeat measure designs). Regardless, menstrual cycle data (or an attempt to collect) should be reported in all heat-related research. Calendar tracking offers a practical means of collecting menstrual cycle data and should be considered as a minimum requirement. For a comprehensive understanding of testing female participants across the menstrual cycle, we refer readers to the detailed guidelines provided by Janse de Jonge et al. (2019). Importantly, as the potential confounding influence of menstrual cycle phase on thermoregulatory-focused performance research can be accounted for (via study design or analytical approach), it is no longer a justifiable excuse to exclude females from participant cohorts (Mazure and Jones, 2015; Freeman et al., 2017; James et al., 2023; Meyer and Cobley, 2024).

#### 4.3.3 Hormonal contraceptives

During the pill-taking phase of the OCP cycle, temperature thresholds for cutaneous vasodilatation, sweat onset and basal T_core_ remain elevated compared to the non-pill taking phase (Martin and Buono, 1997; Tenaglia et al., 1999; Lei et al., 2019). Nonetheless, the current review did not uncover any evidence to suggest that these physiological changes discernibly affected performance in females taking monophasic or triphasic OCPs (Lei et al., 2019; Sims et al., 2007). This is likely because behavioural adjustments (i.e., self-pacing) mitigated these thermoregulatory physiological differences (Lei et al., 2019). Moreover, the magnitude of heat stress associated with the environmental conditions in these studies (i.e., higher humidity) was likely a more significant determinant of exercise performance than OCP cycle phase (Lei et al., 2019). Thus, based on the limited evidence to date, it appears performance in the heat does not change across the OCP cycle. However, it should be noted that when compared to naturally menstruating individuals, a female’s sudomotor response and thermo-sensitivity is attenuated with chronic OCP use (Lei et al., 2019). This could impose a greater thermoregulatory strain during the quasi-luteal phase and potentially alter subsequent performance (Tenaglia et al., 1999). Further research is needed on how OCP and other hormonal contraceptives affect performance in the heat.

Although ∼30% of studies reported including hormonal contraceptive users (*k* = 13/41) in their cohort, only three studies specifically investigated the effect of OCP use compared to other female cohorts or across the OCP month. Continued research into the potential impacts of hormonal contraceptives is necessary given their widespread use among elite athletes (Martin et al., 2018), military personnel (Enewold et al., 2010) and the general population (Pasvol et al., 2022). Moreover, only four studies reported the type of hormonal contraceptive used by participants and the pill taking day/phase that performance tests were conducted in. In keeping with the menstrual cycle phase considerations outlined above, hormonal contraceptive use should be reported in appropriate detail when characterising female participants. For further guidance see Elliott-Sale et al. (2021).

### 4.4 Age

Whilst females typically have a lower $\dot{V}$ O_2max_ than males (Santisteban et al., 2022), the reduction with age is similar between sexes; declining by ∼1% per year after 30 years of age (Huggett et al., 2005). Given the increased cardiovascular strain during exercise in a hot environment (Arngrímsson et al., 2003), aerobic capacity (i.e., $\dot{V}$ O_2max_) remains a prerequisite for successful performance in hot conditions (Bassett and Howley, 2000).

In addition, during perimenopause and menopause the reproductive sex hormone oestrogen declines and levels fluctuate (Santoro, 2016). Subsequently, hormonal replacement therapy (HRT) is commonly prescribed to manage menopausal symptoms, with both menopause and HRT influencing body temperature regulation (Tankersley et al., 1992; Brooks et al., 1997). Only 28% of participants were aged 41–60 years. Furthermore, we did not identify any studies specifically investigating performance in groups of peri-menopausal, menopausal, or post-menopausal females (aged 40–60 years). More masters athletes (aged > 40 years) are participating in endurance and ultra-endurance events (>6 h), with a higher ratio of female-to-male athletes in such competitions (Knechtle et al., 2012; Lepers et al., 2013). There is also comprehensive evidence of an ageing workforce (Barakovic Husic et al., 2020). Therefore, peri- and post- menopausal females are increasingly at risk of exertional heat stress. Further research is needed to understand how menopause affects female aerobic capacity, performance and susceptibility to heat-related injuries and illness so that females can work and perform safely and optimally throughout their lifespan.

### 4.5 Work performance

Our current knowledge of physiological responses and work capacity in hot conditions relies heavily on data from males (Corbett et al., 2023). Males typically present with higher absolute and relative sweat rates, which increases their capacity for evaporative heat loss (Gagnon and Kenny, 2011). Furthermore, males typically present with a higher $\dot{V}$ O_2max_ (Santisteban et al., 2022). Therefore, when undertaking an absolute workload, females are required to work at a higher relative intensity than their male counterparts (Havenith, 2001). Alongside these differences, political factors (i.e., inadequate policies and economic disparities), and cultural expectations (i.e., traditional roles and clothing choices) increase the susceptibility of females to occupational heat stress (Chauhan & Kumar, 2016; WHO, 2017). It is therefore concerning that only three studies identified by the current review included females within their cohorts or specifically investigated work performance/productivity in females (Sen et al., 1983; Vincent et al., 2018; Tenaglia et al., 1999). Furthermore, only one study was conducted in a developing country context (Sen et al., 1983), where workers are more vulnerable to the impacts of global warming and heat waves (Huyer, 2016; Sheahan and Barrett, 2014). No studies to date have investigated the effect of body armour or protective clothing on female performance.

### 4.6 Interventions

Sex differences in HA adaptations have been previously identified, with females requiring longer (i.e., 10-days rather than 5-days HA; Mee et al., 2015) or more intense (i.e., 2-h long vs. 60–90 min; Mee et al., 2018) HA stimuli to gain phenotypic HA adaptations. Our scoping review included only one HA study that showed in females nine, but not 4 days of HA improved time-trial performance in the heat (Kirby et al., 2019). Given the known physiological adaptations that occur with HA, it is also surprising that 18/41 included studies did not report participants’ HA status prior to testing. Reporting such characteristics should be considered fundamental to the interpretation of physical performance under heat stress.

Cooling intervention studies (Zimmermann et al., 2017; Hunter et al., 2006; Arngrïmsson et al., 2004a; Taylor et al., 2014) showed limited evidence that cooling improved female *or* male performance in hot conditions. However, in male cohorts ice slurry and ice vest interventions have been shown to improve performance in the heat (Ückert and Joch, 2007; Quod et al., 2008; Kay et al., 1999). Due to higher surface area-to-mass and lower lean body mass ratios, ice slurry and vest interventions may aid heat dissipation in females more than males (Havenith, 2001; Sharma and Kailashiya, 2016). Hence, more research tailored specifically to the female population is warranted.

Dehydration (>2% body mass loss) leads to a reduction in plasma volume, which increases cardiovascular and thermoregulatory strain and can subsequently impair aerobic performance (Cheuvront et al., 2010). Females have a lower total body water volume and whole blood volume than males (Ritz et al., 2008), and a lower proportion of their total body water is distributed in the extracellular compartment. Together these factors reduce the volume of fluid available for sweating during exercise. Thus, heat stress combined with dehydration could further exacerbate thermoregulatory sex differences. Yet only two studies in the current review examined the effect of dehydration, with females forming just part of the investigated cohort (as opposed to an independent cohort; Slater et al., 2005; Casa et al., 2010).

### 4.7 Performance: female vs. male

Although more studies are including mixed cohorts in their study design, we found a dearth of literature (*k* = 5) investigating sex differences and performance in hot conditions. Currently, thermoregulatory plus performance and exercise-based research is still predominantly male-focused (Hutchins et al., 2021; Meyer and Cobley, 2024). Considering the vast number of females engaging in physically demanding tasks in the heat, further exploration of potential sex differences will assist with tailoring female-specific recommendations.

### 4.8 Quality of the data

The sample size of females within studies was relatively small. The mean sample size for female vs. female comparisons was *n* = 10 (range: 4–162) and for female vs. male comparisons the mean sample size was *n* = 13 (range: 5–88). Twenty-eight studies did not provide a power calculation or justify their sample sizes and therefore, these may have been underpowered. The aim of this review was to scope the evidence. Several considerations should be made with regards to the limited sample sizes, level of available evidence and limited number of studies that met the inclusion criteria.

### 4.9 Future directions

Of the included studies, female participants on average had an excellent-to-superior fitness level ( $\dot{V}$ O_2max_: mean 48.9, range: 35.0–60.0 mL/kg/min; Dourado et al., 2021). Cardiorespiratory fitness significantly enhances an individual’s heat tolerance and performance capacity in uncompensable heat (McLellan, 2001). As such, further investigation is warranted to determine the influence of training status and/or cardiorespiratory fitness on heat tolerance and performance in the heat within female cohorts. This could be particularly relevant for female workers required to wear protective clothing.

There is an urgent need for comprehensive data on occupational heat strain in females and its cumulative effect on productivity and female’s livelihoods. In low- and middle-income countries where females make up a significant proportion of the workforce (both within formal and informal sectors; United Nations, 2015), such information is imperative for understanding the impact of occupational heat stress. Further research is needed to support evidence-based policy changes, as well as inform work-place intervention or education-based programmes (Lucas et al., 2022; Razzak et al., 2022).

This review identified a dearth of research investigating strategies to mitigate the negative effects of heat strain in females. Future research should focus on the feasibility of large-scale interventions to mitigate the burden of heat stress for both male and female personnel/athletes. Given that HA is the most potent stimulus for phenotypic adaptations to improve heat tolerance and performance in the heat, further research is warranted to optimise HA in female cohorts. Furthermore, understanding the HA decay profile in females (and if there are sex differences) would provide valuable insights regarding the optimal timing of HA protocols for females prior to competition, military operations or seasonal agricultural work. Whilst acknowledging the challenges of conducting traditional HA protocols (i.e., 10 consecutive HA days) in females and standardising menstrual cycle phase in pre-to-post HA testing, menstrual cycle phase/hormonal contraceptive use should still be tracked and reported in female participants to aid data interpretation. It is also worth noting that emerging HA approaches (Mee et al., 2018; Kirby et al., 2021) show promise for females aiming to acclimate before performing in the heat. For further insights, we recommend consulting a comprehensive review by Kelly et al. (2023), which provides a framework for designing and executing heat adaptation strategies specifically tailored to the needs of females.

Hormonal variations across the menstrual cycle influence fluid regulation (Stachenfeld and Keefe, 2002; Giersch et al., 2020). Females also typically have lower sweat rates and electrolyte losses than males (Armstrong et al., 2016). Thus, current rehydration guidelines, which are largely based on data from males, may exceed the requirements for females (Speedy et al., 2001). Again, further research in this area is needed.

Pregnant females were excluded from the current study due to the unique demands/risks heat stress poses for pregnant females. There is increasing recognition that pregnant females (along with their foetus) may be more vulnerable to the effects of excessive/exertional heat stress (Samuels et al., 2022). The American College of Obstetricians and Gynecologists advises pregnant women avoid exertional heat stress (ACOG, 2015). Furthermore, females that are/possibly pregnant are often excluded from heat stress experimental studies due to the perceived risks. How such risks should be managed in occupational settings is unclear/evolving. Therefore, to avoid underserving this complex and important topic, pregnancy was considered beyond the scope of the current study’s research question.

## 5 Conclusion

The increasing number of females performing physically challenging occupations and sports amid rising global temperatures highlights a clear need to understand the potential impacts of heat stress on female performance and work productivity. Despite an increase in the proportion of females participating in performance-related thermoregulatory studies over the last 20 years, females remain largely under-represented in this research area, with alarming gaps in our knowledge of how females are affected during key life-stages (i.e., during menopause) or in the occupational sector. Prioritising the inclusion of females in participant samples should remain a key focus for researchers. Additionally, considering key female participant characteristics (e.g., menstrual cycle/hormonal contraception phase; hormonal contraceptive duration of use and type; menstrual cycle irregularities, etc.) during study design, execution and reporting is essential if performance-based physiological research is to be conducted and interpreted appropriately. Such steps will also aid the development of female-specific research questions. Finally, there is a need to provide female workers and athletes with sound and relevant evidence-based recommendations. This will promote an inclusive environment that not only embraces female participation in physically demanding activities but also supports their health and success in these demanding roles amidst an ever-changing climate.
