# Supplementary material for: Mapping the evidence on the impact of heat stress on exercise and work performance in females: a scoping review
Source: Front Physiol. 2025 Jun 3;16:1507398. doi: 10.3389/fphys.2025.1507398 (PMC12170321; doi:10.3389/fphys.2025.1507398)
Supplement: Supplementary file 1 [file Table1.docx]

Supplementary Material

# Supplementary Data

**Table S.1:** Complete search strategy for each database (literature search updated November 2023).

|  | **PubMed** | **Medline** | **Sport Discus** | **Web of Science** |
| --- | --- | --- | --- | --- |
| 1 | (Female OR Woman OR Menstrual) Mesh and Keyword | (Female OR woman.mp. OR exp Women OR exp Menstrual Cycle OR menstrual.mp).ti,ab,kw | female or women or woman or females or menstrual | ((TS=(female OR women OR menstrual*) )) *AND*DOCUMENT  TYPES:  (Article) AND English |
| 2 | (Heat OR Hyperthermia OR Hot OR Body temperature OR Acclimatisation OR Acclimatization OR Acclimation) Mesh and key word | (heat.mp. OR hot.mp. OR exp Body Temperature OR acclimat*.mp. OR exp Acclimatization OR  *temperature OR  exp Hyperthermia, Induced OR hypertherm*.mp).ti,ab,kw | Hot or heat or heat stress or hyperthermia or body temperature or acclimation or acclimatisation or acclimatization | ((((((TS=(Hot or heat or thermoregulation or hyperthermia* or *temperature or acclimat*) ))))) *AND*LANGUAGE:  (English) *AND* DOCUMENT TYPES:  (Article)  *Indexes=SCI-EXPANDED, SSCI, A&HCI, CPCI-S, CPCI-SSH, BKCI-S, BKCI-SSH, ESCI, CCR-EXPANDED, IC Timespan=All years* |
| 3 | (Athletic OR Performance OR Exhaustion OR Exercise OR Thermotolerance OR Workload OR Productivity OR Occupation OR Work OR Heart rate) Mesh and key word | (exp Athletes OR athletes.mp. OR exp Sports/= OR sport.mp. OR performance.mp. OR exp Athletic Performance OR exp "Task Performance and Analysis" OR exp Work Performance OR exhaustion.mp. OR exp Heat Exhaustion OR exp Exercise OR exercise.mp. OR thermotolerance.mp. OR exp Thermotolerance OR workload.mp. OR exp Workload OR exp Efficiency OR efficiency.mp. OR exp Occupations OR occupation.mp. OR exp Work OR work.mp. OR  "heart rate".mp. OR exp Heart Rate OR  productivity.mp).ti,ab,kw | Athletic or work or heart rate or Occupation or tolerance or productivity or efficiency or performance or workload or thermotolerance or sports or athletes or performance or exercise or physical activity or exhaustion or fatigue or burn out or burnout | ((((((TS=(Athletic or efficiency or fatigue or Sports or Performance or Exercise or tolerance or intolerance or exhaustion or fitness or productivity or occupation or Work or workload or "heart rate") )))))) *AND*LANGUAGE:  (English) *AND* DOCUMENT TYPES:  (Article)  *Indexes=SCI-EXPANDED, SSCI, A&HCI, CPCI-S, CPCI-SSH, BKCI-S, BKCI-SSH, ESCI, CCR-EXPANDED, IC Timespan=All years* |
| 4 | #1 AND #2 AND #3 | (exp animal*/ not humans).sh.ti,ab,kw | 1 AND 2 AND 3 | 1 AND 2 AND 3 |
| 5 | (#4 NOT Animal*) ti.ab | 1 AND 2 AND 3 NOT 4 | 4 Not animal |  |
| 6 | Limit 5 to English | Limit 5 to English language | Limit 4 to English language |  |

**Table S.2:** PICO, selection and inclusion processes

| **PICO** | **Inclusion** | **Exclusion** |
| --- | --- | --- |
| Population | Female participants aged 16-60 years. | Pregnant individuals |
|  | Healthy (i.e. no history of cardiovascular or respiratory diseases, diabetes, obesity, heat illness, spinal cord injury or recent musculoskeletal injury (within the past 6 months)). |  |
| Intervention | Wet bulb globe temperature (WBGT) of $\geq$23 ℃ for the performance trials. | Water-based interventions |
|  | Sustained physical activity/work/exercise lasting ≥5 minutes (including both continuous and intermittent protocols; intermittent bouts must last >30s or 30m) | Cognitive tasks |
|  |  | Resistance based exercise protocols measuring 1 repetition maximum or confined to the upper body. |
| Comparison | Healthy male or female control group or comparison group aged 16-60 years performing a valid performance test. |  |
| Outcome | A physical or productivity outcome to exhaustion, measured as the time to complete a set distance/workload/repetitions; maximal amount of work/exercise completed in a fixed amount of time; or the maximum duration or distance that could be sustained at a submaximal speed or power output. Tests included VO_2max_, time to exhaustion, time trial. | No valid performance measure, for example controlled heat production, heat stress test, heat tolerance test. |
| Study design | Randomised controlled trials, pseudo randomised control trials, observational studies from peer-reviewed, scientific journals and reported in English language with full-text available. | Case studies, review articles, conference abstracts, unpublished research and study protocols. |

**File S.1:** Data Charting Protocol

*General information*

Title, lead author, year published, country

Study characteristics:

Methods, aims of study, hypothesis, Study design (e.g. randomised control study, pseudo randomised control study, non-randomised control study, cohort study, cross sectional study, case control study)

Design (e.g. parallel, repeated measures).

Type of comparison (e.g. female vs female; female vs male; hot vs hot, hot vs cool)

Statistical methods

Study procedure

Protocol for intervention

Type of intervention, if appropriate (e.g. clothing, heat acclimation, fluid intake)

Details of intervention

Participant Characteristics

Total, age, height, body mass, BMI, VO_2max_

Female group characteristics (e.g. menstrual phase/pill taking day; eumenorrheic/ type of contraceptives)

Are participants heat acclimated at baseline?

Performance Test

Protocol for performance test

Mode of exercise (e.g. running, cycling)

Stopping criteria

Time of year

Time of day

Location

Any dropouts?

Environmental Conditions

Ambient temperature (mean, maximum, minimum; °C); relative humidity (mean, maximum, minimum; %); WBGT (mean, maximum, minimum; °C); dew point (Mean, minimum, maximum; °C)

*Performance Outcomes*

Time Trial in Hot Conditions

Time (min, sec); speed (m/s); power output (mean, maximum; W), distance (km).

Time to Exhaustion in Hot Conditions

Time (min, sec), distance (km), power output (mean, maximum; W).

VO_2max_ in Hot Conditions

VO_2max_, power output (maximum; W), Time (min, sec).

Work productivity

Work (kJ), time (min, sec), speed (mean, maximum; m/s), power output (mean, maximum; W).

*Thermoregulatory outcomes*

Core Temperature Measurement

Description of procedures, rest/initial/onset (°C), mean during exercise/work (°C), peak (°C), delta (°C).

Skin Temperature Measurement

Description of procedures, rest/initial/onset (°C), mean during exercise/work (°C), peak (°C), delta (°C).

Sweat Rate/Sweat Loss/Change in Body Mass

Description of procedures, was any fluid ingested during the trial, sweat rate (L/hour), sweat rate body surface area (g/h^2^/m^2^), sweat loss/body mass loss (L/kg), body mass change (%).

Core to Skin Gradient

Description of procedures, rest/initial/onset (°C), mean during exercise/work (°C), peak (°C), delta (°C).

*Cardiovascular/cardiometabolic outcomes*

Heart Rate

Description of procedures, rest/initial/onset (BPM), mean during exercise/work (BPM), peak (BPM), delta (°C), % HR max (%).

Plasma Volume

Description of procedures, pre (mL), post (mL), % change (%).

Blood Lactate

Description of procedures, rest/initial/onset (mmol/L), mean during exercise/work (mmol/L), peak (mmol/L), delta (mmol/L), % change (%).

Oxygen Consumption

Description of procedures, rest/initial/onset, mean during exercise/work, peak, delta.

RER

Description of procedures, rest/initial/onset, mean during exercise/work, peak, delta.

Cardiac Output

Description of procedures, rest/initial/onset (L/min), mean during exercise/work (L/min), peak (L/min), delta (L/min).

Stroke Volume

Description of procedures, rest/initial/onset (L/min), mean during exercise/work (ml/beat), peak (ml/beat), delta (ml/beat).

Skin blood flow

Description of procedures, rest/initial/onset, mean during exercise/work, peak, delta.

*Perceptual Measures*

RPE

Description of procedures, rest/initial/onset, mean during exercise/work, peak, delta.

Thermal Comfort

Description of procedures, rest/initial/onset, mean during exercise/work, peak, delta.

Thermal Sensation

Description of procedures, rest/initial/onset, mean during exercise/work, peak, delta.

Thirst

Description of procedures, rest/initial/onset, mean during exercise/work, peak, delta.

*Conclusions*

Main outcomes, limitations, future directions.

**Table S.3:** Level of Evidence Criteria for Included Studies

| **Characteristics** | **Level 1 (High):** | **Level 2 (Moderate):** | **Level 3 (Low):** |
| --- | --- | --- | --- |
| **Type of study** | Participants are recruited and randomly assigned into an intervention group. The outcome of the intervention is assessed. | A research study that follows groups of individuals over time, who are alike in many ways but differ by a certain characteristic.  A study that observes two groups of individuals. One group has the condition you are interested in (the cases) and one group does not have it (the controls). | Cross sectional, uncontrolled study  design, such as case Studies (individual or very small cohort).  Analysing a group of people who already have certain characteristics (retrospective). |
| **Study Design** | A control group is required; pre-/post- or repeated-measures design was used. | Pre-/post- and/or repeated measures design was used.  A retrospective design may have been used.  A control or comparison group may have been used, but was not required | Post-test only or cross-sectional design was used. A retrospective design may be used. A control group is not required. |
| **Allocation into groups** | Randomised group allocation only. | Groups were not required to be randomised | Groups were not required to be randomised |
| **Type of study** | Randomised Control Trial | Cohort/ Prospective Cohort, Retrospective Cohort, Case-Control studies | Cross Sectional Study |

**File 2:** Quality of Evidence Tool

1. Are the aims/research questions clearly stated in the main text?

Did the authors describe their goal in conducting this research? Is it easy to understand what they were looking to find?

2. Did the authors describe their goal in conducting this research? Is it easy to understand what they were looking to find?

Does the study design address all aspects of the research questions (Y = ideal study design e.g., RCT; P = there is a more suitable study design; N = wrong study design for this question)

3. Does the study design address all aspects of the research questions (Y = ideal study design e.g., RCT; P = there is a more suitable study design; N = wrong study design for this question)

Did the authors describe the group of people from which the study participants were selected or recruited, using demographics?  Was the location stated, and time period (e.g. time of year data was collected and time period data was collected over)?  Time of year/seasonality is important due to the effect of time of year on outdoor temperatures.  Time period is important due to the effect it can have on repeated heat exposures.  If you were to repeat this study, would you know who to recruit, from where, and when?

4. Is the sample size justified? (e.g. power analysis)

Did the authors present their reasons for selecting or recruiting the number of people included or analyzed? Do they note or discuss the statistical power of the study?

For example, a paragraph in the methods section may explain the sample size needed to detect a hypothesized difference in outcomes. This may be justified in the limitations section of the discussion.

5. Is there a detailed account of participants recruitment and allocation to experimental groups?

Participant recruitment is the act of defining, finding and inviting representatives of your target audience into your user research or usability study. The methods of recruitment should be clearly documented. Details of how this occurred should be included, so the methods of recruitment ad allocation could be repeated. For example, "For allocation of the participants, a computer-generated list of random numbers was used."

If details of recruitment of participants is explained (e.g. participants were recruited via poster advertisements) and explanation of how they were then allocated into group is included (e.g. random number generator) then this would score yes. If only one of these is mentioned score partly. No if neither are reported.

6. Are there clearly defined inclusion/ exclusion criteria for participants?

Were the inclusion and exclusion criteria clearly identified? Were the same underlying criteria used for all of the subjects involved?

7. Are reasons for non-participation/ drop-out described?

Describe the completeness of outcome data for each main outcome, including attrition and exclusions from the analysis. State whether attrition and exclusions were reported, the numbers in each intervention group (compared with total randomized participants).

8. Are all outcome measurement outcomes described in sufficient detail in order to repeat this study?

Yes, an explanation is provided to explain the piece of equipment or questionnaire used to obtain each outcome measure. This would mean that you would be able to obtain the piece of equipment/use the equation/questionnaire provided to repeat the study.

E.g. devices used to measure core body temperature. (temperature probe model and make).

If core temperature has been estimated using a specific formula and details are provided so you would be able to obtain these values yourself this would count as yes.

Type of metabolic cart, details of calibration, sampling.

 Partly, some outcome measures are not reported in enough detail/ are missing.

 No, many outcome measures are not outlined in enough detail preventing replication.

9. Is there a rationale for the choice of data collection tools for the paper's key outcomes?

Yes, a clear rationale is provided explaining measurement tool selection for key outcome measures.

For example validation or citing other key papers in the research area.

 Partly, some explanation is provided.

 No, no rationale/explanation provided.

10. Are the key outcome measures the most appropriate to answer the research question?

Yes, key outcome measures are measured directly using appropriate measures the most reliable and valid for the experimental setting.

 Partly, key outcomes measures are estimated from calculations as opposed to directly measured. Perhaps some direct measurement could be possible in the experimental setting.

No, key outcome measures could have been measured directly but haven't been.

11. Does the statistical/data analysis fit the research question?

Yes, observational study: confidence intervals but no P values necessary.

Experimental study: P values and effect sizes are reported.

Partly, appropriate statistical analysis but some information missing i.e. effect size, P values.

No, multiple t-tests aren't corrected for.

More sophisticated statistical models could have been used.

Inappropriate reporting of stats.

12. Were potential confounding variables measured and assessed during analyses?

Were key potential confounding variables measured and adjusted for, such as by statistical adjustment for baseline differences? Logistic regression or other regression methods are often used to account for the influence of variables not of interest.

E.g. type of clothing, menstrual phase, fluid consumption.

13. Were potential confounding variables measured and assessed during analyses?

Were key potential confounding variables measured and adjusted for, such as by statistical adjustment for baseline differences? Logistic regression or other regression methods are often used to account for the influence of variables not of interest.

E.g. type of clothing, menstrual phase, fluid consumption.

**Table S.4:** Summary of acclimation status, time of year/day, dropouts, interventions, location, exercise modality, study procedures, performance tests and performance outcomes for all included studies.

| **Study** | **Acclimation status** | **Time of year** | **Time of day** | **Dropouts** | **Intervention** | **Location** | **Modality** | **Performance test** | | **Performance outcomes** | |
| --- | --- | --- | --- | --- | --- | --- | --- | --- | --- | --- | --- |
| Aylwin 2023 | 63% performed 5 - 30 days HA before the event | Summer | 23:30-05:00 | Not reported | No intervention | Field-road race | Race walking | 50-km | TT | Time (min)  % of PB | Females:  293.9 ±21.8 min  113 ±7% |
|  |  |  |  |  |  |  |  |  |  |  | Males:  263.0 ±12.1 min  116 ±4% |
| Risa Iwata 2021 | No prior heat exposure | Not reported | 13:00-16:00 | Not reported | No intervention | Lab-environmental chamber | Cycling ergometer | 55% V̇O_2max_ until exhaustion | TTE | Time (min) | Females:  42.8 ±9.6 min |
|  |  |  |  |  |  |  |  |  |  |  | Males:  41.8 ±8.0 min |
| Zheng  2021 | No prior heat exposure  (>1 month) | Spring; Autumn; Winter | Before 09:00 | Excluded: anovulatory cycle (*n*=3); iron supplementation (*n*=1), incompletion (*n*=1) | No intervention | Lab-environmental chamber | Cycling ergometer | 20-min fixed intensity followed by 30-min TT | TT | Work (kJ)  PO (W) | Hot:  272 ±59 kJ  151 ±33 W |
|  |  |  |  |  |  |  |  |  |  |  | Temp:  280 ±57 kJ  156 ±32 W |
| Tan  2021 | Not reported | Winter | 04:30 | Not reported | No intervention | Field-road race | Running outdoors | 2017 Standard Chartered Singapore Marathon | TT | Time (min)  speed (m/s) | Females:  163 ±6 min  4.3 ±0.1 m/s |
|  |  |  |  |  |  |  |  |  |  |  | Males:  147 ±4 min  4.8 ±0.1 m/s |
| Racinais 2021 | Not reported | Summer | 23:30 | Incompletion (n = 7; 2 females, 5 males) | No intervention | Field-road race on flat loops | Race walking | 2019 World Athletics 50 km | TT | Time (h:min:s)  % of PB | Females:  5:04:17 ±0:23:13 h:min:s;  117.7 ±5.4% |
|  |  |  |  |  |  |  |  |  |  |  | Males:  4:22:28 ±0:11:09 h:min:s  114.3 ±3.8% |
| Racinais 2022 | 48% of athletes had a dedicated 5–30-day heat preparation period. | Summer | 23:30 | Incompletion (n = 22; 6 females, 16 males) | No intervention | Field-road race on flat loops | Race walking | 2019 World Athletics Championships 50 km | TT | % of PB | Females:  111 ±4% |
|  |  |  |  |  |  |  |  |  |  |  | Males:  115 ±5% |
| Gavel  2021 | No prior heat exposure  (>4 weeks) | Winter | Same time of day | None reported | Menthol mouth rinse | Lab-environmental chamber | Cycling ergometer | 30-km | TT | Time (h:min:s)  PO (W) | Menthol mouth rinse: 1:02:34 ±5:40 h:min:s  142 ±32 W |
|  |  |  |  |  | Placebo mouth rinse |  |  |  |  |  | Placebo:  1:04:02 ±4:54 h:min:s  134 ±27 W |
| Janse de Jonge 2012 | Testing took place in the cooler months to avoid natural HA | Autumn / Winter | Same time of day | In Hot, two participants did not complete the sub-maximal exercise protocol.  Excluded due to low progesterone levels (n=3). | No intervention | Lab-environmental chamber | Cycling ergometer | 60-min steady-state (60% V̇O_2max_) followed by incremental TTE | TTE | Time (min) | Hot:  Luteal 63.6 ±10.4 min  Follicular 66.7 ±8.1 min |
|  |  |  |  |  |  |  |  |  |  |  | Temp:  Luteal 71.9 ±4.2 min Follicular 72.8 ±3.0 min |
| Hashimoto 2014 | Not reported | | Morning | Not reported | Carbohydrate beverage | Lab-environmental chamber | Cycling ergometer | 90-min cycling (50% V̇O_2_peak) followed by a TT | TT | Time (min:s) | Carbohydrate:  Luteal 11:18 ±3:00 min:s  Follicular 11:32 ±2:36 min:s |
|  |  |  |  |  | Placebo beverage |  |  |  |  |  | Placebo:  Luteal 11:21 ±2:27 min  Follicular 11:15 ±2:43 min:s |
| Racinais 2019 | Not reported | October in Quatar (Autumn) | Not reported | | No intervention | Field-flat terrain | Cycling outdoors | 2016 UCI Road World Championships 40 km Team TT | TT | PO (W)  PO (W/kg) | Females:  40.8 ±0.7 W  4.5 ±0.2 W/kg |
|  |  |  |  |  |  |  |  |  |  |  | Males:  39.2 ±0.2 W  4.8 ±0.2 W/kg |
| Kirby  2019 | No prior heat exposure  (>2 months) | February, April, May, and June (UK) | Same time of day (within 2-h) | Excluded due to relocation  (n=1) | 9-days HA (baseline vs post HA) | Lab-environmental chamber | Cycling ergometer | 15-min | TT | Speed (m/s)  PO (W)  Distance (km) | PRE-HA:  31.9 ±2.3 m/s  180 ±34 W  7.96 ±0.58 km |
|  |  |  |  |  |  |  |  |  |  |  | POST-HA:  32.8 ±2.4 m/s  195 ±36 W  8.2 ±0.59 km |
| Wright  2002 | No prior heat exposure  (>3 months) | Not reported | Same time of day | Not reported | No intervention | Lab-environmental chamber | Running treadmill | 30-min | TT | Distance _absolute_ (km)  Distance _adjustedlean bodymass_ (km)  Distance _adjusted bodymass_ (km) | Follicular:  4.9 ±0.13 km  5.93 ±0.42 km  4.24 ±0.43 km |
|  |  |  |  |  |  |  |  |  |  |  | Luteal:  4.74 ±0.20 km  5.65 ±0.44 km 4.03 ±0.38 km |
|  |  |  |  |  |  |  |  |  |  |  | Males:  5.2 ±0.42 km  3.47 ±0.70 km  6.53 ±0.65 km |
| Slater  2005 | Testing took place in the winter months to avoid natural HA | Winter | Same time of day | Not reported | Fluid restriction: 4% BML in the preceding 24-h | Lab-environmental chamber | Rowing ergometer | 2,000 m | TT | Time (s) | Females Fluid restriction:  Hot: 461.4 ±11.2 s  Temp 457.2 ±9.3 s |
|  |  |  |  |  |  |  |  |  |  |  | Females Control:  Hot: 457.9 ±10.1 s  Temp: 453.7 ±10 s |
|  |  |  |  |  | Control: No weight limit specified |  |  |  |  |  | Males Fluid restriction:  Hot:403.3 ±7.8 s  Temp 400.3 ±7.4 s |
|  |  |  |  |  |  |  |  |  |  |  | Males Control:  Hot:403.0 ± 6.0 s Temp_:_398.2 ± 7.4 s |
| Taylor  2014 | No prior heat exposure  (>2 months) | Winter | Same time of day | Not reported | Precooling (20- min cold-water shower) | Lab-environmental chamber | Rowing ergometer | 2,000 m | TT | Time (min)  PO (W) | Hot Pre-cooling:  8.52 ±0.24 min  143 ±5 W |
|  |  |  |  |  |  |  |  |  |  |  | Hot control:  9:01 ±0:31 min  133 ±6 W |
|  |  |  |  |  |  |  |  |  |  |  | Temp control:  8.48 ±0.25 min  141 ±5 W |
|  |  |  |  |  | Control: 20-min passive rest |  |  |  |  |  |  |
| Sen  1983 | Not reported | Not specifically stated (plucking season is April-November) | 8am - 4pm | Not reported | No intervention | Field-tea plantation | Plucking shoots | Hand movements. Shoots plucked. | Productivity | Work (kJ/min)  speed (shoots/min)  speed (hand movements/min) | Fast pluckers:  17.3 ±7.5 kJ/min  141 ±44 shoot/min  143 ±27 hand movements/min |
|  |  |  |  |  |  |  |  |  |  |  | Slow pluckers:  14.04 ±4.17 kJ/min  101 ±31 shoots/min  128 ±17 hand movements/min |
| Millard-Stafford 1995 | 5-weeks training in hot, humid conditions | Summer | Morning | Not reported | No intervention | Field-8x 5-km segments | Running outdoors | 40-km | TT | Time (min) | Females:  183.8 ±4.2 min |
|  |  |  |  |  |  |  |  |  |  |  | Males:  173.5 ±8.5 min |
| Kupchak 2017 | NR | Summer (August in Texas) | 07:00 am | Not reported | No intervention | Field-road race on largely flat terrain | Cycling outdoors | 164-km | TT | Time (h) | Females:  7.29 ±1.09 h |
|  |  |  |  |  |  |  |  |  |  |  | Males:  6.42 ± 1.07 h |
| Lei  2019 | No prior heat exposure  (>1 month) | Spring; Autumn | Same time of day (±1h) | Not reported | No intervention | Lab-environmental chamber | Cycling ergometer | 30-min | TT | PO (W)  Work capacity (kJ) | Hot humid:  143 ±16 W (unaffected by OCP phase)  258 ±28 kJ (similar for OCP) |
|  |  |  |  |  |  |  |  |  |  |  | Hot Dry: 152 ±16 W (unaffected by OCP phase)  273 ±29 kJ (similar for OCP) |
| Lei  2017 | No prior heat exposure  (>1 month) | Spring; Autumn; Winter | Same time of day (±1-h) | Excluded due to progesterone level <9.5nmoll-1 (n=3) | No intervention | Lab-environmental chamber | Cycling ergometer | 30-min | TT | Work capacity  (kJ) | Humid: 248 ±40 kJ  Dry: 263 ± 39 kJ  EF: 257 ±37 kJ  ML: 255 ±43 kJ |
|  |  |  |  |  |  |  |  |  |  |  |  |
| Ftaiti  2010 | Not reported | | Morning | Not reported | No intervention | Lab-environmental chamber | Cycling ergometer | 60% PPO | TTE | Time (min) | Hot:  46.4 ±10 min |
|  |  |  |  |  |  |  |  |  |  |  | Temp: 70.4 ±14 min |
| Adams  2017 | Not reported | Summer | Not reported | | No intervention | Field-road race | Running outdoors | 2014 Falmouth 7-mile | TT | Time (min)  Speed (min/km) | Females:  57.57 ±5.53 min  5.07 ±0.31 min/km |
|  |  |  |  |  |  |  |  |  |  |  | Males:  53.34 ±8.23 min  4.44 ±0.44 min/km |
| Arngrimsson  2004a | Accustomed to exercising in the heat | Not reported | Same time of day | Not reported | Preheating: 20-min active warm-up (33% V̇O_2max_) | Lab-environmental chamber | Running treadmill | V̇O_2max_ test | TTE & V̇O_2max_ | Time (min)  V̇O_2_ (l/min) | Females Hot:  _Pre-heating_ 9.05 ±1.79 min;  2.52 ±0.4 l/min  _Control:_ 11.38 ±1.43 min; 2.75 ±0.44 l/min |
|  |  |  |  |  |  |  |  |  |  |  | Females Temp:  _Pre-heating:_ 12.64 ±1.45 min  3.01 ±0.44 l/min  _Control_: 13.00 ±1.3 min;  3.01 ±0.45 l/min |
|  |  |  |  |  | Control: no warm-up |  |  |  |  |  | Males Hot:  _Pre-heating:_ 8.77 ±1.31 min  3.75 ±0.48 l/min  _Control:_ 11.51 ±1.15 min  4.13 ±0.51 l/min |
|  |  |  |  |  |  |  |  |  |  |  | Males Temp:  _Pre-heating:_ 13.15 ±1.41min  4.52 ±0.47 l/min  _Control:_ 13.01 ±1.18 min  4.52 ±0.46 l/min |
| Arngrimsson  2004b | Accustomed to exercising in a hot environment | Not reported | Same time of the day | Not reported | Ice: Cooling vest with ice packs during warm-up | Lab-environmental chamber | Running treadmill | 38-min warm-up, followed by 5-km TT | TT | Time (min) | Females:  Ice: 20:55 ±1:07 min  Placebo: 21:06 ±1:01 min |
|  |  |  |  |  | Placebo: Regular T-shirt during warm-up |  |  |  |  |  | Males  Ice 17:07 ±1:01 min  Placebo 17:20 ±1:01 min |
| Cordery 2017 | Not accustomed to exercise in a warm environment | Not reported | Morning | Not reported | Bupropion | Lab-environmental chamber | Cycling ergometer | 60% V̇O_2_peak for 60-min, followed by 30-min TT | TT | Total work (kJ) | Bupropion:  291 ±46 kJ |
|  |  |  |  |  | Placebo |  |  |  |  |  | Placebo:  269 ±46 kJ |
| Armstrong 2016 | Not reported | | Morning | N/A as retrospective sampling | No intervention | Field-road race flat terrain | Cycling outdoors | 164-km | TT | Time (h) | Females:  7.74 ±1.25 h |
|  |  |  |  |  |  |  |  |  |  |  | Males:  7.23 ±1.76 h |
| Suvi  2017 | No prior heat exposure  (>2 months) | Not reported | 1-h after breakfast, i.e. 8:00 am. | Not reported | Caffeine | Lab-environmental chamber | Walking on treadmill | Walk | TTE | Time (min) | Females:  Caffeine 76 ±11 min  Placebo 82 ±15 min |
|  |  |  |  |  | Placebo |  |  |  |  |  | Males:  Caffeine 83 ±17 min  Placebo 82 ±14 min |
| Zimmermann  2017 | Tested in the cooler months to avoid HA | Same time of day | | Not reported | Ice: 30-min rest ingesting crushed ice | Lab-environmental chamber | Running outdoors | 800-kJ work | TT | Time (min)  PO (W) | Ice:  62:47 ±7:45 min  215.9 ±27.8 W |
|  |  |  |  |  | Control: 30-min rest ingesting water |  |  |  |  |  | Control:  64:11 ±7:29 min  211.1 ±26.1 W |
| Armstrong 2012 | Not reported | Summer | 08:00-15:00 | Not reported | No intervention | Field-road race on flat terrain | Cycling outdoors | 164-km | TT | Time (h) | Females:  7.77 ±1.24 h |
|  |  |  |  |  |  |  |  |  |  |  | Males:  7.23 ±1.75 h |
| Horstman 1982 | Not reported | | | | No intervention | Lab-environmental chamber | Cycling ergometer | 40% V̇O_2max_ up to 120-min | TTE | Time (min) | Females:  84 ±8 min |
|  |  |  |  |  |  |  |  |  |  |  | Males:  75 ±10 min |
| Hunter  2006 | Not reported | | | | Ice vest: Raced in Nike Ice-Vest | Field-cross-country race | Running outdoors | 5 km | TT | Time (min:s ±s) | Ice vest:  18:15 ±0:43 min:s |
|  |  |  |  |  | Control: raced in typical race clothing |  |  |  |  |  | Control:  18:27 ±00:57 min:s |
| O'Neal  2012 | Not reported | Summer (July to Late August in America) | morning or evening | 5 dropped out due to injuries | No intervention | Field-most of the course was asphalt | Running outdoors | 1 hr | TT | Speed (min/km)  Distance (km) | Females:  5.95 ±0.67 min/km  10.0 ±1.04 km |
|  |  |  |  |  |  |  |  |  |  |  | Males:  5.40 ±0.61 min/km  11.1 ±1.11 km |
| Beal  2022 | Not reported | Doha: September 2019 | Not reported | ∼41% dropout Doha | No intervention | Field- road race | Running outdoors | Doha marathon vs London marathon | TT | Time (h:min:s) | Doha:  2:51:51 ±0:11:26 h:min:s |
|  |  | London: August 2017 |  | ∼15% dropout London |  |  |  |  |  |  | London:  2:41:19 ±0:10:00 h:min:s |
| Tenaglia 1999 | Winter to avoid natural HA | January to Early June | 08:00 | None | No intervention | Lab-environmental chamber | Repeats of 15-min walking, 15-min rest | Up to 300-min | TTE | Time (min) | OCP users:  q-EF 113.0 ±5.8 min  qML 116.8 ±11.2 min |
|  |  |  |  |  |  |  |  |  |  |  | Non-users:  EF 128.1 ±13.4 min  ML 107.4 ±8.6 min |
| Jenkins 2023 | Unacclimated | Not reported | | None | No intervention | Lab-environmental chamber | Cycling ergometer | 20-km | TT | Time (min)  PO (W) | Females Hot:  43.7 ±2.8 min  125.5 ±19.9 W |
|  |  |  |  |  |  |  |  |  |  |  | Females Temp:  34.0 ±2.5 min  157.0 ± 23.9 W |
|  |  |  |  |  |  |  |  |  |  |  | Males Hot:  37.77 ±3.83 min  186.96 ± 42.92 W |
|  |  |  |  |  |  |  |  |  |  |  | Males Temp:  34.45 ±4 min  238.9 ±60.0 W |
| Casa  2010 | Partial HA, based on the running history questionnaire | Not reported | 13:00 | NR | Dehydrated: fluid restriction 22-h before race. | Field-3 x 4-km loops | Running outdoors | 12-km | TT | Time (min) | Females:  Dehydrated: 62.6 min  Control: 58.8 min |
|  |  |  |  |  | Control: fluids ad libitum. |  |  |  |  |  | Males:  Dehydrated: 49.6 min  Control: 48.1 min |
| Andrade 2023 | Not specifically HA. | Not reported | Morning, afternoon or evening | None | No intervention | Lab-environmental chamber | Running treadmill | 10-km | TT | Speed (km/h) | Females:  11.9 km/h |
|  |  |  |  |  |  |  |  |  |  |  | Males:  11.2 km/h |
| Vincent 2018 | Not reported | | 06:00 | Not reported | No intervention | Lab-heaters | Work circuits | 55-min | TT | Blackout hose (reps)  Charged hose advance (reps)  Lateral repositioning (reps)  Rake (reps) | Females:  158 reps  86 reps  576 reps  23 reps  4 reps |
|  |  |  |  |  |  |  |  |  |  |  | Males:  180 reps  118 reps  646 reps  21 reps  6 reps |
| Wallace 2017 | Not reported | | | None | No intervention | Lab-environmental chamber | Cycling ergometer | 30-min (60% PPO), 30-min rest, followed by TTE (80% PPO) | TTE | Time (min) | Females:  7.1 ±2.6 min |
|  |  |  |  |  |  |  |  |  |  |  | Males:  8.9 ±2.9 min |
| Sims  2007 | Winter to control for HA | June-September (southern hemisphere) | Morning | Not reported | High sodium beverage | Lab-environmental chamber | Cycling ergometer | 70% V̇O_2peak_ | TTE | Time (min) | High sodium:  96.3 ±25.4 min |
|  |  |  |  |  | Low sodium beverage |  |  |  |  |  | Low sodium:  77.6 ±26.3 min |
| Hosokawa 2016 | Not reported | Summer | Morning | 2 participants were excluded (passed the gastrointestinal pill before race) | No intervention | Field -road race | Running outdoors | 11.3 km warm-weather road race | TT | Time (min) | Females:  57.4 ±7.5 min |
|  |  |  |  |  |  |  |  |  |  |  | Males:  54.6 ±7.5 min |
| Stone 2021 | Not reported | | | | No intervention | Lab- environmental chamber | Cycling ergometer | 15-min or 45-min followed by V̇O_2max_ test | V̇O_2max_ | V̇O_2max_ (mL/kg/min)/ maximal PO (W_max_) | 15-min:  Follicular: 43.8 ±8.5 mL/kg/min;  216 ±51 W_max_  Luteal: 44.0 ± 10.4 mL/kg/min;  224 ± 49 W_max_ |
|  |  |  |  |  |  |  |  |  |  |  | 45-min:  Follicular: 37.8 ±6.9 mL/kg/min;  186 ±40 W_max_  Luteal: 36.9 ± 8.6 mL/kg/min;  191 ± 50 W_max_ |

Values are for female vs. female comparisons, unless a male comparison is stated. Values are for hot vs. hot comparisons unless a temperate comparison is reported. Abbreviations: PB, personal best; BML, body mass loss; PO, power output; PPO, peak power output; V̇o_2max_; maximal oxygen consumption; TT, time trial; TTE, time to exhaustion; HA, heat acclimation; OCP, oral contraceptive pill; EF, early follicular; ML, mid-luteal; qEF, quasi early follicular; qML; quasi mid-luteal; NR, not reported; Temp, temperate.

**Table S.5:** Detailed summary of all participant characteristics for all included studies in this review.

| **Study** | **Group** | **N** | **Age (years)** | **Body mass (kg)** | **Height (cm)** | **V̇O_2max_ (ml/kg/min)** | **Menstrual state of females**  **(NM, HC, E)** | **Brand/ type of HC reported (yes/not reported/n/a)** | **Exogenous hormone concentrations reported (Yes/not reported/n/a)** | **Menstrual phase (including days after menses if reported)** | **Verification of Menstrual Cycle Phase** |
| --- | --- | --- | --- | --- | --- | --- | --- | --- | --- | --- | --- |
| Aylwin  2023 | F | 8 | 30 ±5 | 54.4 ±6.3 | 167 ±6.4 | Not reported | Not reported  n/a | | | | |
|  | M | 19 | 33 ±7 | 67.9 ±5.8 | 180.7 ±6.1 | Not reported |  |  |  |  |  |
| Risa Iwata 2021 | F | 12 | 22 ±2 | 58.2 ±10 | 158.9 ±5.6 | 36.5 ±4.2 | NM | n/a | | L | Not reported |
|  | M | 12 | 25 ±2 | 65.8 ±10.3 | 170.6 ±5.5 | 43.6 ±3.3 |  |  |  |  |  |
| Zheng  2021 | F | 8 | 37 ±7 | 63 ±7 | 166 ±9 | 46.4 ±7.1 | NM | n/a | | ML (day 20 ±2) and 22 ± 3) and EF (day 3 ± 1 and 7 ± 2) | Self-reported and verified via LH, oestrogen and progesterone concentrations |
| Tan  2021 | F | 6 | 31 ±6 | 49.8 ±4.3 | Not reported | | Not reported | | | | |
|  | M | 6 | 28 ±7 | 57.4 ± 4.1 | Not reported | |  |  |  |  |  |
| Racinais  2021 | F | 5 | 29 ±5 | 55.8 ±5.9 | 167 ±6 | Not reported | Not reported | | | | |
|  | M | 17 | 30 ±5 | 67.5 ± 6.5 | 179 ±7 |  |  |  |  |  |  |
| Racinais  2022 | F | 23 | 29 ±5 | 52.4 ±6.8 | 166 ±6 | Not reported | Not reported | | | | |
|  | M | 44 | 32 ±6 | 67.6 ±6.4 | 179 ±7 |  |  |  |  |  |  |
| Gavel  2021 | F | 9 | 27 ±1 | 60.7 ±6.3 | 165 ±5.4 | 50.8 ±6 | HC: monophasic intrauterine device (*n*=7); triphasic OCP (*n*=1), NM (*n*=1). | Yes | Not reported | Endocrine environment was consistent within the participant | Not reported |
|  |  |  |  |  |  |  |  |  |  |  |  |
| Janse de Jonge 2012 | F | 12 | 24 ±4 | 67.8 ±9.8 | 165.8 ±4.2 | 40 ±6.9 | NM | n/a | | F (day 3–6) and L (day 19–25). | Verification via oestrogen and progesterone concentrations |
| Hashimoto 2014 | F | 6 | 23 ±3 | 48.7 ±6.1 | 156.2 ±2.4 | 39.5 ±5.3 | NM | n/a | | F and L | Calendar tracking and verification via oestrogen and progesterone concentration |
| Racinais  2019 | F | 4 | 27 ±4 | 58 ±6 | 169 ±5 | Not reported | Not reported | | | | |
|  | M | 7 | 29 ±3 | 74 ±5 | 184 ±6 |  |  |  |  |  |  |
| Kirby  2019 | F | 8 | 27 ±5 | 61 ±5 | 170 ±4 | 47 ±6 | HC: OCP (*n*=2), implant (*n*=3), intrauterine device (*n*=1). NM (*n*=2) | Yes | Not reported | Did not control for menstrual cycle | Not reported |
|  |  |  |  |  |  |  |  |  |  |  |  |
| Wright  2002 | F | 5 | 21 ±1 | 60.2 ±0.6 | 168 ±2.35 | 51 ±1.9 | HC: triphasic OCP (*n*=3). NM (*n*=2) | Not reported | | F and L | Calendar tracking (preceding 3 months) |
|  | M | 5 | 25 ±2 | 80.8 ±4.3 | 184.4 ±3.8 | 59.2 ±3.8 |  |  |  |  |  |
| Slater  2005 | F | 9 | 23 ±4 | 63.2 ±2.6 | 171.7 ±5 | 55 ±3.1 | HC: Monophasic OCP >4 weeks. | Not reported | | Not reported | Verification via oestrogen and progesterone concentrations |
|  | M | 8 | 22 ±4.0 | 74.2 ±1.3 | 183.2 ±1.8 | 64.5 ±2.5 |  |  |  |  |  |
| Taylor  2014 | F | 8 | 21 ±2 | 66.8 ±3.1 | 166.1 ±4 | Not reported | Not reported | | | F (*n*= 5), L (*n*= 3) | Not reported |
| Sen  1983 | F | 12 | 26 | 40.1 | 149.1 | Not reported | Not reported | | | | |
| Millard-Stafford  1995 | F | 6 | 29 ±3 | 53.9 ±1 | 168.9 | 60 ±3.1 | NM (*n*=4). OCP (*n*=2). | Not reported | | L (*n*=5). Not tested on same day of MC. | Self-reported questionnaire and interview data |
|  | M | 6 | 31 ±2 | 69.5 ±3.2 | 186.7 ±2.1 | 69.2 ±1.6 |  |  |  |  |  |
| Kupchak  2017 | F | 9 | 46 ±9 | 68.8 ±13.3 | 166 ±10 | Not reported | Not reported | | | | |
|  | M | 28 | 54 ±9 | 83.3 ±11.9 | 176 ±9 |  |  |  |  |  |  |
| Lei  2019 | F | 10 | 25 ±5 | 68 ±10 | 167 ±5 | 55 ±9 | HC:(Monophasic OCP >1 year. | y | Not reported | L and F (day 3–5 and 18–20 or 10–12 and 25–27) | Verification via oestrogen and progesterone concentration |
|  |  |  |  |  |  |  |  |  |  |  |  |
| Lei  2017 | F | 13 | 34 ±9 | 64 ±6 | 165 ±5 | 58 ±9 | NM | n/a | n/a | EF (day 3 and 6) and ML (day 18 and 21) | Verification via oestrogen and progesterone concentration |
|  |  |  |  |  |  |  |  |  |  |  |  |
| Ftaiti  2010 | F | 7 | 23 ±1 | 63 ±4 | 170 ±5 | 35 ±4 | Not reported | | | MF (day 4- 8) | Not reported |
| Adams  2017 | F | 16 | 36 ±10 | 59.8 ±7.1 | Not reported | Not reported | Not reported | | | | |
|  | M | 16 | 40 ±12 | 76.3 ±8.5 |  |  |  |  |  |  |  |
| Arngrimsson 2004b | F | 11 | 24 ±4 | 56 ±4.9 | 164.8 ±5.7 | 53.9 ±7.5 | NM (*n*=8), OCP (*n*=3). | Not reported | | | |
|  | M | 11 | 23 ±5 | 70.1 ±8.8 | 178.2 ±4.4 | 64.7 ±5.3 |  |  |  |  |  |
| Arngrimsson 2004a | F | 8 | 22 ±2 | 55.9 ±4.3 | 167.7 ±5.5 | 58 ±3.2 | Not reported | | | | |
|  | M | 9 | 23 ±4 | 67.7 ±4.2 | 178.6 ±4.4 | 66.7 ±5.9 |  |  |  |  |  |
| Cordery  2017 | F | 9 | 21 ±2 | 64.6 ±6 | 168 ±8 | 51 ±7 | NM | n/a | | F (day 10) | Self-reported |
| Armstrong 2016 | F | 15 | 43 ±7 | 64.6 ±7.3 | 166 ±5 | Not reported | Not reported | | | | |
|  | M | 88 | 44 ±9 | 86.4 ±12.3 | 179 ±7 |  |  |  |  |  |  |
| Suvi  2017 | F | 10 | 23 ±2 | 61 ±5.4 | 167.3 ±5.2 | 45.6 ±4 | HC: OCP | Not reported | | | |
|  | M | 13 | 25 ±4 | 78.8 ±7.9 | 182.7 ±5.5 | 51.7 ±2.7 |  |  |  |  |  |
| Zimmermann 2017 | F | 10 | 28 ±6 | 68 ±11.5 | 167.6 ±6.5 | Not reported | HC (monophasic OCP >3 months) | Not reported | Yes | Not reported | |
| Armstrong  2012 | F | 6 | 41 ±4 | 67.3 ±7.2 | 168 ±2.9 | Not reported | Not reported | | | | |
|  | M | 42 | 38 ±6 | 85.9 ±14.8 | 179.7 ±7.1 |  |  |  |  |  |  |
| Horstman 1982 | F | 4 | 21 ±0 | 66.8 ±3.9 | 166.5 ±4.1 | Not reported | Not reported | | | | |
|  | M | 6 | 22 ±1 | 77 ±3.5 | 176.2 ±3.6 |  |  |  |  |  |  |
| Hunter  2006 | F | 18 | 20 ±2 | 57 ±2.3 | 168 ±6 | Not reported | Not reported | | | | |
| O'Neal  2012 | F | 20 | 41 ±9 | 61.1 ±7.6 | 164 ± 6 | 52.3 ±7.2 | Not reported | | | | |
|  | M | 19 | 41 ±12 | 77 ±5.6 | 177 ±6 | 61.2 ±8.8 |  |  |  |  |  |
| Beal  2022 | F ((Doha) | 92 | 30 ±5 | Not reported | Not reported | Not reported | Not reported | | | | |
|  | F (London) | 70 | 30 ±5 |  |  |  |  |  |  |  |  |
| Tenaglia  1999 | F | 9 | 23 ±2 | 60.4 ±3 | 165 ±3 | 44.8 ±2.6 | NM | n/a | n/a | EF (days 2-5), ML (days 19-22) | Verified with oestrogen and progesterone concentrations |
|  | F | 9 | 23 ±1 | 64.5 ±1.5 | 166 ±3 | 43.6 ±2.7 | OCP; monophasic (*n*=7), triphasic (*n*=2) | Yes | Yes | Quasi EF (days 2-5), Quasi ML (days 19-22) |  |
| Jenkins  2023 | F | 7 | 25 ±6 | 63.6 ±7.7 | 165.1 ±5.5 | 48.8 ±5.4 | Not reported | | | L for NM or weekly during the active pill phase if OCP user | Not reported |
|  | M | 7 | 32 ±17 | 71.3 ±3.3 | 175.3 ±3.8 | 60.5 ±7.3 |  |  |  |  |  |
| Casa  2010 | F | 8 | 27 ±4 | x | 164 ±7 | Not reported | Not reported | | | | |
|  | M | 9 | 27 ±9 | x | 176 ±5 |  |  |  |  |  |  |
| Andrade  2023 | F | 6 | x | 59.8 | Not reported | 46.2 | HC >12 months | Yes | Not reported | Active phase of the OCP (Day 2-21) | Not reported |
|  | M | 40 | x | 78.65 |  | 57.1 |  |  |  |  |  |
| Vincent  2018 | F | 4 | 40 | 73 | 166 | Not reported | Not reported | | | | |
|  | M | 14 | 35 | 92.2 | 181 |  |  |  |  |  |  |
| Wallace  2017 | F | 4 | 34 | 62.9 | 170.7 | 57.5 | Not reported | | | | |
|  | M | 14 | 40 | 77 | 177.5 | 61.1 |  |  |  |  |  |
| Sims  2007 | F (hormonal contraceptive) | 6 | 24 ±5 | 61.9 ±5.2 | 160.6 ±6 | 53.1 ±2.5 | Tri-phasic OCP > 6 months | Not reported | | day 18-20 | Verification via oestrogen and progesterone concentrations |
|  | F (naturally menstruating) | 7 | 29 ±6 | 62.7 ±4.8 | 165.2 ±5 | 51.2 ±1.8 | NM | Not reported | n/a | day 20-21 |  |
| Hosokawa 2016 | F | 15 | 37 ±10 | 59.3 ±7.1 | Not reported | Not reported | Not reported | | | | |
|  | M | 15.00 | 41 ±11 | 77.2 ±8 |  |  |  |  |  |  |  |
| Stone  2021 | F | 7.00 | 24 ±5 | 58.3 ±9.3 | 164.7 ±5.8 | 42.90 | NM (*n*=4), HC (*n*=3) | Yes | Not reported | F (day 2-5); LP (day 19-22) | Self-reported and verified via oestrogen and progesterone concentrations |

Abbreviations: OCP, Oral contraceptive pill; HC, hormonal contraceptive; NM, naturally menstruating; F, follicular; L, luteal; EF early follicular; ML mid luteal; Y, yes; n, no; *n,* number of participants.

**Table S.6:** Table for environmental conditions of all included studies in the review.

| **Study** | **Environmental conditions** | | | **Location** |
| --- | --- | --- | --- | --- |
|  | **Temperature (°C)** | **Humidity (%)** | **WBGT (°C)** |  |
| Aylwin  2023 | Females: 32.7 ±0.2 | Females: 76 ±3 | Females: 28.2 ±0.9 | Field – 50-km race walk |
|  | Males: 31.1 ±0.5 | Males: 76 ±3 | Males: 28.2 ±0.9 |  |
| Iwata  2021 | 38 | 40 | 30* | Lab-environmental chamber |
|  |  |  |  |  |
| Zheng  2021 | 32.00 ±0 (Hot) | 53 ±8 (Hot) | 29 (Hot)* | Lab-environmental chamber |
|  | 20.0 ±1 (temp) | 75 ±3 (Temp) | 16 (Temp)* |  |
| Tan  2021 | Range 27.9 - 27.8 | Range 76 - 80 | Range 26.3 - 26.3 | Field - Singapore Road Marathon |
|  |  |  |  |  |
| Racinais  2021 | 31.1 ±0.5 | 75.7 ±3 | 28.2 ±0.9 | Field – 50-km race walk |
|  |  |  |  |  |
| Racinais  2022 | 31.1 ±0.5 | 75.7 ±3 | 28.2 ±0.9 | Field – 50-km race walk |
|  |  |  |  |  |
| Gavel  2021 | 30.2 ±0.6 | 68.6 ±3.2 | 27* | Lab-environmental chamber |
|  | 30.2 ±0.2 | 69.9 ±0.9 |  |  |
| Janse de Jonge 2012 | 32 | 60 | 27.5* | Lab-environmental chamber |
|  | 20 | 45 | 15* |  |
| Hashimoto  2014 | 30 ±2 | 50 ±5 | 24.5* | Lab- environmental chamber |
|  |  |  |  |  |
| Racinais  2019 | 36.9 ±2.8 | 24.6 ±15.6 | 27.1 ±2.4 | Field – 50-km race walk |
|  |  |  |  |  |
| Kirby  2019 | 35 | 30 | 26* | Lab- environmental chamber |
|  |  |  |  |  |
| Wright  2002 | 33 | 60 | 28.5* | Lab- environmental chamber |
|  |  |  |  |  |
| Slater  2005 | 32.4 ±0.4 (Hot) | 60 ±2.7 (Hot) | 27.5 (Hot)* | Lab- environmental chamber |
|  | 21.2 ±0.7 (Temp) | 29 ±4.5 (Temp) | 15 (Temp)* |  |
| Taylor  2014 | 35 (Hot) | 60 (Hot) | 30.5 (Hot)* | Lab- environmental chamber |
|  | 22 (Temp) | 38.8 (Temp) | 16.5 (Temp)* |  |
| Sen  1983 | 31.8 ± 2.6 | 72.2 ±13.8 (range 27.7- 89) | 27.6 ± 1.4 (range 25.5 - 29) | Field - tea plantation |
|  |  |  |  |  |
| Millard-Stafford 1995 | Range 25 - 32 | Range 70 - 82 | Range 23.1- 28.9 | Field. 8x 5-km segments were completed to a total of 40-km. |
|  |  |  |  |  |
| Kupchak  2017 | Not reported | Range 26.8 - 56.5 | Range 22.3 - 34.6 | Field: The 2015 164-km cycling HHH course |
|  |  |  |  |  |
| Lei  2019 | 29.0 ±0.3 (Humid) | 83 ±2 (Humid) | 27 (Humid and Dry) | Lab- environmental chamber |
|  | 34 ±0.2 (Dry) | 42 ±3 (Dry) |  |  |
| Lei  2017 | 29 ±1 (Humid) | 81 ±3 (Humid) | 27 (Humid and Dry) | Lab- environmental chamber |
|  | 34 ±0.2 (Dry) | 41 ± 3 (Dry) |  |  |
| Ftaiti  2010 | 35 ±0.3 (Hot) | 59 ±6 (Hot) | 30.5 (Hot)* | Lab- environmental chamber |
|  | 22 ±0.4 (Temp) | 53 ±8 (Temp) | 18 (Temp)* |  |
| Adams  2017 | 25.3 ±0.6 | 73.9 ±4.1 | 23.7 | Field- 7-mile Great American running road race |
|  |  |  |  |  |
| Arngrimsson 2004b | 45 (Hot) | 50 (Hot and Temp) | 38 (Hot)* | Lab- environmental chamber |
|  | 25 (Temp) |  | 20.5 (Temp)* |  |
| Arngrimsson 2004a | 32 | 50 | 26.5* | Lab- environmental chamber |
|  |  |  |  |  |
| Cordery  2017 | 30.2 ±0.2 | 50 ±1 | 25* | Lab- environmental chamber |
|  |  |  |  |  |
| Armstrong  2016 | 35 | 32-48 | 26.4-29.0 | Field: The Hotter’N Hell Hundred 164-km cycling race |
| Suvi  2017 | 42 | 20 | 29.5* | Lab- environmental chamber |
|  |  |  |  |  |
| Zimmermann 2017 | 34.9 ±0.3 | 49.8 ±3.5 | 29* | Lab- environmental chamber |
| Armstrong  2012 | 34.4 ±5 (range 24.4 - 39.5) | 53 ±14 (range 40 - 83) | 30.8 ±3.5 (Range 22.8 - 33.6) | Field: The Hotter’N Hell Hundred 164-km cycling race |
|  |  |  |  |  |
| Horstman  1982 | 45 | Not reported | 23 | Lab- environmental chamber |
|  |  |  |  |  |
| Hunter  2006 | 26 | 72 | 23.5* | Field - cross-country race |
|  |  |  |  |  |
| O'Neal  2012 | 27 ±2.4 | 79 ±11 | 24.1 ±1.5 | Field: 1-h running TT (laps) |
|  |  |  |  |  |
| Beal  2022 | Doha: 32 | Doha: 77.9 | Doha: 29.6 | Field: Doha marathon |
|  | London: 19 | London: 56 | London: 15.5 | Field: London marathon |
| Tenaglia  1999 | 40 | 30 | 30* | Lab- environmental chamber |
| Jenkins  2023 | 36 | 1.96/3.92 kpa | 27.5/32* | Lab- environmental chamber |
|  | 18 | 1.96 kpa | 18* |  |
| Casa  2010 | Not reported | Not reported | Hydration: 25.3 | Field: 3 x 4-km laps running |
|  | Not reported | Not reported | Dehydration: 27.0 |  |
| Andrade  2023 | 33 | 61 | 28.7 | Lab- environmental chamber |
| Vincent  2018 | 33 | 40 | 26* | Lab- air conditioning units |
| Wallace  2017 | 35 | 50 | 29* | Lab- environmental chamber |
| Sims  2007 | 32 | 50 | 26.5* | Lab- environmental chamber |
| Hosokawa  2016 | 25.3 ±0.6 | 74.1 ±4.1 | 23* | Field- 11.3-km road race |
| Stone  2021 | 35 | Not reported | Not reported | Lab- environmental chamber |

Abbreviations: WBGT; wet bulb globe temperature; TT; time trial. *, WBGT calculated using https://www.climatechip.org
